# Induction of SOX17 with stimulation of WNT, TGF‐beta, and FGF signaling drives embryonal carcinomas into the yolk‐sac tumor lineage resulting in increased cisplatin resistance

**DOI:** 10.1002/ijc.35385

**Published:** 2025-03-02

**Authors:** Mara Kotthoff, Margaretha A. Skowron, Felix Bremmer, Fatma Parmaksiz, Pia Kretschmer, Alexa Stephan, Alexander Fichtner, Tobias Lautwein, Katharina Raba, Janina Fuß, Karl Köhrer, Daniel Nettersheim

**Affiliations:** ^1^ Department of Urology, Urological Research Laboratory, Translational UroOncology, Medical Faculty and University Hospital Düsseldorf Heinrich Heine University Düsseldorf Düsseldorf Germany; ^2^ Institute of Pathology University Medical Center Göttingen Göttingen Germany; ^3^ Genomics and Transcriptomics Laboratory (GTL), Biomedical Research Center Heinrich‐Heine‐University Düsseldorf Düsseldorf Germany; ^4^ Institute for Transplantation Diagnostics and Cell Therapeutics, Core Facility Flow Cytometry, Medical Faculty and University Hospital Düsseldorf Heinrich Heine University Düsseldorf Düsseldorf Germany; ^5^ Competence Center for Genomic Analysis University Hospital Schleswig‐Holstein Kiel Germany; ^6^ Center for Integrated Oncology Aachen Bonn Cologne Düsseldorf (CIO ABCD) Germany

**Keywords:** cisplatin resistance, differentiation, embryonal carcinoma, SOX17 / FOXA2, yolk‐sac tumors

## Abstract

Relapsing germ cell tumor (GCT) patients often harbor components of the aggressive subtype yolk‐sac tumor (YST), suggesting that YST formation is an escape mechanism under therapy. Nevertheless, the molecular mechanisms inducing YST development from its stem cell‐like precursor embryonal carcinoma (EC) are largely unexplored. We demonstrated that the induction of the transcription factor SOX17 together with the stimulation of WNT, TGF‐beta / Activin, and FGF signaling drives EC cells into the YST lineage. Single cell RNA sequencing revealed that this cell fate switch was accompanied by the upregulation of the typical YST factors *AFP*, *ANKRD1*, *APOA1*, *CST1*, *FOXA2*, *GATA6*, and *GPC3* and microRNAs, while pluripotency‐related genes *NANOG*, *POU5F1*, and *SOX2* were downregulated. Chromatin immunoprecipitation followed by sequencing analysis revealed that SOX17 may act in concert with FOXA2 and GATA factors to initiate YST formation. Xenografting of the YST‐like cells into nude mice led to the growth of mixed GCT with YST components, confirming that these cells are able to form a YST in vivo. Moreover, the expression of cisplatin resistance factors was induced in a subpopulation of YST‐like cells, suggesting that the formation of a YST is accompanied by the acquisition of cisplatin resistance. Indeed, the YST‐like cells presented as less sensitive to cisplatin than their parental cells. Our study deciphered the molecular mechanisms forcing EC to differentiate into the YST lineage, which is accompanied by the acquisition of cisplatin resistance, confirming that YST formation is an escape mechanism for GCT under therapy. Thus, GCT patients should be screened for YST elements under therapy to identify patients at risk of developing therapy resistance.

## Graphical abstract

What's new?

Yolk‐sac tumor formation has been suggested to be an escape mechanism during therapy in patients with relapsing germ cell tumors. The molecular mechanisms inducing yolk‐sac tumor development from stem cell‐like precursor embryonal carcinomas remain unclear. This study suggests that activation of SOX17 together with stimulation of WNT, TGF‐beta / Activin and FGF signaling drives embryonal carcinomas into the yolk‐sac tumor lineage. This cell fate switch is accompanied by acquisition of cisplatin resistance, confirming yolk‐sac tumor formation as an escape mechanism. Patients with germ cell tumors under therapy should be screened for yolk‐sac tumor elements to identify those at risk of developing resistance.

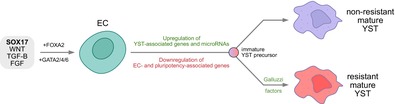

## 1 INTRODUCTION

Germ cell tumors (GCT) mainly affect young men between the ages of 14 and 44 and are the most common solid tumor malignancies in this age group, with incidence increasing steadily, particularly in Western countries. However, GCT can also be found in women, i. e., in the ovary.^1^, ^2^ GCTs can be divided into the seminomas (SEM) and non‐SEM, which both arise from a germ cell neoplasia in situ as a result of a defective primordial germ cell (PGC).^1^, ^3^ The SEM are latent pluripotent and resemble PGC with regard to morphology, gene expression, and epigenetics, whereas the totipotent to pluripotent embryonal carcinomas (EC) represent the stem cell‐like population of the non‐SEM.^1^ EC have the potential to differentiate into tumors of all three germ layers (teratoma) or into extraembryonic tissues, such as choriocarcinoma and yolk‐sac tumors (YST).^1^, ^4^

Besides orchiectomy, cisplatin‐based chemotherapy and radiation therapy remain the standard of care for GCT.^5^, ^6^ The overall cure rate of GCT patients is high, except for patients facing the aggressive GCT subtype YST, which is associated with a poor prognosis. Relapsing GCT patients often develop therapy‐resistant YST components, suggesting that YST formation is an escape mechanism for GCT under therapy.

Previously, we highlighted the pioneer transcription factors FOXA2 and SOX17, which are both involved in endodermal differentiation processes, as key factors in YST biology, inducing expression of YST‐specific genes, like AFP, ANKRD1/6, APOA1/A2/B, CST1, GATA3/4/6, and GPC3.^7^, ^8^ Upregulation of these YST‐associated genes is accompanied by downregulation of pluripotency‐ / EC‐ / PGC‐related factors.^8^ Furthermore, YST shows high activity of TGF‐beta, MAPK, and WNT signaling compared to EC.^8^ Thus, induction of the pioneer transcription factors SOX17 and FOXA2, as well as activation of differentiation‐promoting signaling cascades, seems to be an indispensable step for initiating YST formation, which is then accompanied by inactivation of the pluripotency network.

As YST are aggressively growing malignant tumors, finding a way to treat YST specifically and improving diagnostics requires special attention. Despite this clinical need, the molecular mechanisms triggering YST development and conferring cisplatin resistance remain largely unexplored. Thus, this publication attempts to unwire the complex molecular mechanisms driving YST development to gain a deeper understanding of the disease itself, identify developmental stages to interfere with to prevent the formation of YST from EC, and deduce therapeutic targets specifically for YST.

## 2 MATERIALS AND METHODS

### 2.1 Standard laboratory techniques

A description of standard laboratory methods, like RNA / protein isolation, production of lentiviral particles, quantitative q(RT‐)PCR, immunohistochemistry, western blotting, flow cytometry‐based apoptosis analysis and cell sorting, XTT assays, and xenotransplantation, is given in the “Supplemental Material and Methods” section in Data S2.

### 2.2 Cell culture

All cell lines were provided and cultivated as described in Table S1A and as previously published.^9^, ^10^ All human cell lines have been authenticated using “short tandem repeats” (STR) profiling within the last three years. All experiments were performed with mycoplasma‐free cells.

### 2.3 In vitro differentiation of EC cells into the YST lineage

For differentiation into a YST‐like cell fate, 1 × 10^5^ EC‐MPHv2^+^ cells (stably expressing the MS2‐P65‐HSF1 activator helper complex for the CRISPR/dCas9‐SAM system) were seeded out into a 10 cm cell culture dish in 6 mL of standard culture medium. For each experiment, a solvent control was included. The next day, fresh medium containing polybrene (1 : 1000, Merck, Darmstadt, Germany) and 450 μL lentiSAMv2‐*SOX17* lentiviral particles was added to induce endogenous *SOX17* expression. After 20 h, cells were washed, and YST‐like differentiation medium (standard culture medium + 100 ng/mL ActivinA, 3 μM CHIRON, 25 ng/ml FGF2, 1 μg/mL heparin) was added and renewed every 48 h (Table S1B).

### 2.4 microRNA sequencing

Library preparation for microRNA sequencing (microRNAseq) was done with the “NEXTFLEX Small RNA‐Seq Kit v4” (Revvity, Hamburg, Germany) according to manufacturer's protocol with 200 ng input.^11^ Paired‐end sequencing (2× per sample) was done on a NovaSeq6000 SP flow cell using the “NovaSeq 6000 SP Reagent Kit” (v1.5). Demultiplexing was done with “bcl2fastq” tool (v2.20.0.422). Analysis was performed using “nf‐core/smrnaseq” (v2.2.0) against the human reference genome GRCh38. The statistical quality control was performed using the “FastQC” tool, generating the mean quality scores of all sequences, sequence duplication levels, and the count of unique / duplicate reads. Counts per million were calculated by the “trimmed mean of M values” (TMM) normalization method in the “edgeR” package (v4.0).^12^, ^13^ The sequencing coverage and quality statistics for each sample are summarized in Table S2A.

### 2.5 Single cell RNA sequencing

Single cells were generated on the “10× Chromium Controller” system for single‐cell droplet library generation utilizing the “Chromium Next GEM Single Cell 3' Kit v3.1” (10x Genomics, Pleasanton, CA, USA) according to manufacturer's instructions. Single‐cell‐RNA‐sequencing (scRNAseq) was carried out on a “NextSeq2000” system (Illumina Inc. San Diego, USA) with a mean sequencing depth of 50,000 reads / cell. Raw sequencing data was processed using the 10x Genomics “CellRanger” (v7.0) software. Raw “BCL” files were de‐multiplexed and processed to “FASTQ” files (“CellRanger mkfastq”). Alignment of reads to the GRCh38 genome and “Unique Molecular Indentifier” (UMI) counting was performed (“CellRanger count”) to generate a gene‐barcode matrix. All samples were aggregated and normalized for sequencing depth (“CellRanger aggr”). Further analyses were carried out with the “Seurat v4.1.1” R package.^12^, ^14^, ^15^, ^16^, ^17^ Quality control consisted of removal of cells with < 200 detected genes and of genes expressed in less than three cells. Cells with a mapping rate of > 10 % to the mitochondrial genome were removed. Cell doublets have been removed from the dataset (“DoubletFinder v2.0”).^18^ Normalization has been carried out utilizing “SCTransform” (v2.0).^19^, ^20^ Dimensional data reduction was achieved by principal component analysis based on identified variable genes and subsequent “uniform manifold approximation and projection” (UMAP) embedding. Cells were clustered using the graph‐based clustering approach implemented in “Seurat” (v4.1.1). Markers defining each cluster and differential gene expression between different clusters were calculated by using a “Wilcoxon Rank Sum” test (in “Seurat”). The trajectory graph was constructed in “Monocle3” (v 1.3.7) and the cells were ordered in pseudotime.^21^, ^22^, ^23^ For detecting genes changing as a function of pseudotime, the graph was sub‐setted into the two developmental pathways leading to resistant and non‐resistant mature YST‐like cells, before running the “graph_test” function. The sequencing coverage and quality statistics for each sample are summarized in Table S2B.

### 2.6 Chromatin‐immunoprecipitation sequencing

For chromatin‐immunoprecipitation (ChIP), 1 × 10^7^ cells per immunoprecipitation (IP) were seeded into 15 cm dishes in quadruplicates. Upon two washing steps with PBS, DNA–protein cross‐linking was performed by adding 80 μL of “ChIP cross‐link Gold” (Diagenode, Seraing, Belgium) to 20 mL PBS containing 1 mM MgCl_2_ for 30 min. Cells were washed twice with PBS before continuing with another cross‐linking step using formaldehyde (Sigma‐Aldrich, Taufkirchen, Germany) and the “SimpleChIP Plus Enzymatic Chromatin IP Kit” (Cell Signaling Technology, Leiden, The Netherlands) according to the manufacturers protocol. To fragmentize the chromatin, 0.5 μL micrococcal nuclease per IP was added and samples were sonicated 3× for 20 s followed by a pause of 30 s at an amplitude of 80 %. One IP of the quadruplicates was used to purify DNA and to validate the chromatin digestion and concentration as a quality control. 2 % of input control was extracted and the ChIP was continued according to the manufacturers protocol by incubating 10 μg of antibody / IP over night at 4 °C on a Hula mixer at 20 rpm. Upon IP using “Protein G” magnetic beads, chromatin was eluted and reversed cross‐linked before purification of DNA. ChIP sequencing (ChIPseq) was performed of IP samples (*n* = 3) and input controls (*n* = 2) according to the standard procedure at Novogene Corporation (Munich, Germany). The quantity and quality of purified DNA were measured by using the “Qubit DNA Assay Kit” in “Qubit 3.0 Fluorometer” (Life Technologies, CA, USA) and the “NanoPhotometer spectrophotometer” (IMPLEN, CA, USA). The library was constructed by “Novogene Corporation,” and quality was monitored on the “Agilent Bioanalyzer 2100” system before being pair‐end sequenced on an Illumina platform. For data analysis, raw reads were processed using the “fastp” software (v 0.19.11).^24^ Clean reads were generated by removing reads containing adapter, reads containing poly‐N, and low‐quality reads. High‐quality and clean reads were aligned to the “Ensembl 110 *Homo sapiens* GRCh38” reference genome using “BWA mem” (v 0.7.12). The “MACS2” peak calling software (v 2.1.0) was used to identify enriched regions of the IP compared to the background (*q*‐value < .05).^25^ The “Homer” software was used to detect de novo sequence motifs, while the “ChIPseeker” allowed to retrieve and annotate peak‐related genes.^26^, ^27^ For visualization of ChIPseq peaks, triplicates were merged using the “Integrative Genomics Viewer.”^28^, ^29^ The sequencing coverage and quality statistics for each sample are summarized in Table S2C.

### 2.7 Online software tools

“The Cancer Genome Atlas” GCT cohort was analyzed using cBioPortal.^30^, ^31^ “ShinyCell for R” was used to analyze and illustrate single cell RNAseq data.^32^ The “STRING” algorithm was used to predict protein–protein interactions.^33^ The “DAVID” algorithm was used to predict molecular functions of gene sets.^34^ “Venny 2.1” was used to generate Venn diagrams. “shinyCircos‐V2.0” and “BoxPlotR” were utilized to visualize ChIPseq data.^35^, ^36^ Enrichment of sequence motifs was graphically illustrated using “ImageGP.”^37^

## 3 RESULTS

### 3.1 Deciphering the mechanisms of YST formation in vitro

**FIGURE 1:**
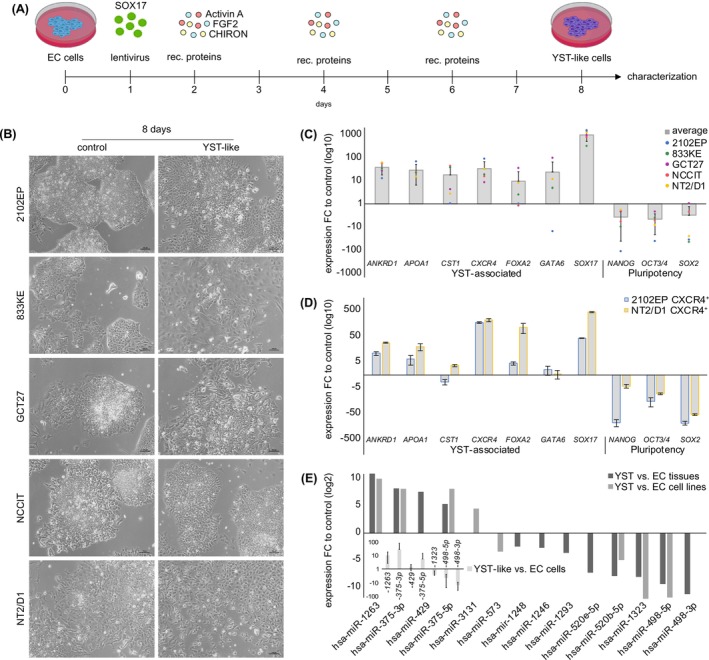
(A) Treatment scheme and experimental design. *SOX17* expression was induced by the CRISPR/dCas9‐SAM system the day after seeding in standard culture medium (d0). WNT, ActivinA, and FGF activators were added on d2 to standard culture medium and was refreshed every 48 h for 8d in total. (B) Morphology of YST‐like cells after SACF treatment on d8 in comparison to the parental cells (solvent control). (C) Expression fold change (FC) of YST‐associated genes (*ANKRD1*, *APOA1*, *CST1*, *CXCR4*, *FOXA2*, *SOX17*) and pluripotency genes (*NANOG*, *OCT3/4*, *SOX2*) in YST‐like cells (from 2102EP, 833KE, GCT27, NCCIT, NT2/D1) on d8 of SACF treatment, normalized to their respective solvent control. Five biological replicates (2102EP, 833KE, GCT27, NCCIT, NT2/D1) were analyzed in technical triplicates each. *GAPDH* and *Beta‐Actin* served as housekeepers and for data normalization. Standard deviations are given above each bar. (D) Expression FC of YST‐associated genes (*ANKRD1*, *APOA1*, *CST1*, *CXCR4*, *FOXA2*, *SOX17*) and pluripotency genes (*NANOG*, *OCT3/4*, *SOX2*) in YST‐like CXCR4^+^ FACS sorted cells (2102EP, NT2/D1) on d8 of SACF treatment, normalized to CXCR4^−^ cells. Two biological replicates (2102EP, NT2/D1) were analyzed in technical triplicates each. *GAPDH* and *Beta‐Actin* served as housekeepers and for data normalization. Standard deviations are given above each bar. (E) microRNAs deregulated in expression in YST versus EC tissues / cell lines based on microRNA sequencing and correlation to data by Wruck et al.^8^ Three YST and EC tissues, as well as two YST cell lines (GCT72, 1411H) and three EC cell lines (2102EP, NCCIT, NT2/D1) were analyzed and sequenced in technical duplicates. Inlay: qPCR validation of selected microRNA expressions in YST‐like versus parental EC cells (average of 2102EP, NCCIT, NT2/D1, GCT27). Four biological replicates (2102EP, NCCIT, NT2/D1, GCT27) were analyzed in technical triplicates each. *hsa‐miR‐16‐5p*, *‐20a‐5p*, *‐25*, *‐30*, and *‐103a‐3p* served as housekeepers and for data normalization. Standard deviations are given above each bar.

Previously, we identified the transcription and endodermal‐differentiation factor SOX17 as a key determinant of the YST cell fate and demonstrated increased expression of WNT, BMP, MAPK, and FGF signaling molecules in YST compared to EC tissues.^8^ Additionally, a previous study identified TGF‐beta as a potent inducer of differentiation of SEM cells into the mixed non‐SEM lineage with YST features (maintained *SOX17* expression, upregulation of *AFP* and *GATA2*/*4*/*6*).^38^ Thus, we decided to induce endogenous expression of the YST fate key factor *SOX17* by the CRISPR/dCas9‐SAM system in combination with stimulation of various signaling pathways to narrow down the optimal conditions for differentiation of EC in the YST lineage.^8^ In five EC cell lines (2120EP, NCCIT, NT2/D1, GCT27, 833KE), we verified an efficient induction of *SOX17* over 96 h on the RNA and protein levels by qRT‐PCR and western blot analysis, respectively (Figure S1A,B). Next, we tested recombinant proteins or inhibitors applied on an everyday basis in NCCIT cells for 4 days (d), leading to activation (a) or inhibition (i) of WNT (a: WNT3a, WNT5, CHIRON, i: XAV‐939), BMP (a: BMP2, BMP4; i: NOGGIN), MAPK (i: SCH772984), TGF‐beta (a: ActivinA) or FGF (a: FGF2 + heparin) signaling. Afterwards, we screened for expression of marker genes differentially expressed between EC and YST (Figure S1C,D). Based on upregulation of expression of YST‐associated genes and downregulation of pluripotency factors, stimulation of WNT (CHIRON), TGF‐beta (ActivinA) and FGF (FGF2 + heparin) signaling seems a promising approach to support YST formation from EC (Figure S1D). Thus, we set up a schedule for differentiation, i. e., at d1, we induced expression of *SOX17* by lentiviral transduction of the CRISPR/dCas9‐SAM components, and from d2 until d8, we additionally applied recombinant proteins or inhibitors every other day (Figure 1A). Subsequently, we tested single, double, triple, and quadruple treatments of CHIRON, ActivinA, and FGF2 in combination with SOX17 induction in NCCIT cells for 8d and screened for changes in gene expression of YST factors afterwards (Figure S1E). Here, the most promising approach for induction of a YST‐like cell fate was the quadruple combination of SOX17 induction with TGF‐beta (ActivinA; 100 ng/mL), WNT (CHIRON; 3 μM) and FGF (FGF2; 25 ng/mL + 1 μg/mL heparin) signaling stimulation (SACF) (Figure 1A; Figure S1E). Next, we extended our analysis to five EC cell lines in total (2120EP, NCCIT, NT2/D1, GCT27, 833KE). After SACF treatment, the morphology changed considerably from growing in small densely packed colonies to a more loosened polygonal shape (Figure 1B). Additionally, the YST factors *ANKRD1*, *APOA1*, *CST1*, *CXCR4*, *DUSP4*, *FOXA2*, *GATA6*, and *SOX17* were upregulated, while EC and pluripotency factors *NANOG*, *OCT3/4*, and *SOX2* were downregulated in the treated EC cell lines (Figure 1C).^39^

Of note, FGF2 enhanced downregulation of the pluripotency factors *NANOG* and *OCT3/4* and upregulation of some of the tested YST factors (Figure S1D). Additionally, YST‐like cells appeared morphologically more healthy with the addition of FGF2. In a previous study, we found downregulation of FGF2/4 in YST compared to EC tissues. Thus, stimulation of FGF signaling seems to be of benefit at early stages of differentiation, but not necessary for the maintenance of a YST‐like cell fate.

### 3.2 Validation I: Enrichment of YST‐like cells by CXCR4

To further confirm that SACF application induces a YST cell fate, we enriched the YST‐like cells resulting from differentiation (at d8) of 2102EP and NT2/D1 EC cells by CXCR4‐based fluorescence‐activated cell sorting (FACS), which we found strongly upregulated in YST‐like cells and YST.^39^ The cells for CXCR4‐FACS were highly viable with 96 % for YST‐like cells and 98 % for undifferentiated 2102EP / NT2/D1 cells (Figure S1F). A qRT‐PCR analysis demonstrated that the expression of typical YST markers was increased, while the expression of EC and pluripotency markers was reduced in CXCR4^+^ versus CXCR4^−^ cells (Figure 1D).

### 3.3 Validation II: microRNA analysis

Next, we asked if microRNA expression also changes during the differentiation of EC cells to a YST‐typical profile. Therefore, we performed microRNAseq of EC and YST tissues, as well as EC cell lines (2102EP, NCCIT, NT2/D1) and the YST cell line GCT72. Prior to sequencing, the presence of microRNAs in all samples was verified by qPCR analysis of housekeeper microRNAs *hsa‐mir‐16‐5p*, *‐20a‐5p*, *‐25*, *‐30*, *‐103a‐3p*, which were highly and homogeneously expressed in all samples (Figure S1G). To identify YST‐associated microRNAs, we selected all mature microRNAs increased in expression (fold change [FC] > 5) in YST versus EC tissues and cells (Data S1A). Now, we compared these data to microRNAs deregulated in YST versus EC tissues as identified by Wruck et al.,^8^ highlighting 14 microRNAs deregulated (five upregulated; nine downregulated) in YST versus EC tissues or cells (Figure 1E; Data S1A). By qPCR, we validated the up‐ or downregulation of selected microRNAs in YST‐like cells (from 2102EP, NCCIT, NT2/D1, GCT27) compared to the parental EC cells (Figure 1E, inlay). Again, the microRNAs *hsa‐mir‐16‐5p*, *‐20a‐5p*, *‐25*, *‐30*, *‐103a‐3p*, and a *UniSp6*‐spike‐in control were used as housekeepers. The microRNA analysis confirmed a YST‐typical microRNA expression profile in YST‐like cells.

### 3.4 Validation III: Xenografting of YST‐like cells in vivo

**FIGURE 2:**
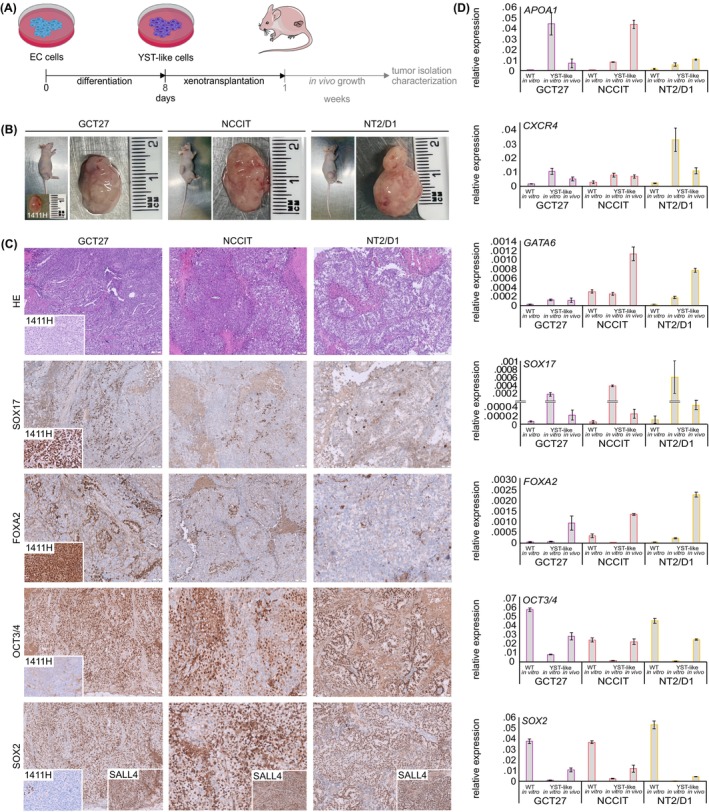
(A) Schedule of xenotransplantation experiments. EC cells were differentiated into the YST‐like lineage and subsequently xenotransplanted into nude mice. After reaching 1.5 cm^3^ in size, tumor tissues were isolated and analyzed. (B) Macroscopic appearance of tumors before and after isolation of xenotransplanted YST‐like cells from GCT27, NCCIT, and NT2/D1 cells or 1411H control cells (at day of isolation). (C) Hematoxylin and eosin and immunohistochemical staining for YST marker proteins FOXA2 and SOX17, the pluripotency factors OCT3/4 and SOX2, and the germ cell marker SALL4 exemplarily for each xenotransplanted YST‐like cell population (GCT27, NCCIT, NT2/D1) and 1411H control cells were performed. Each analysis was conducted in three biological replicates. Scale bars: 100 – 200 μm. (D) Relative gene expression of YST‐associated genes (*APOA1*, *CXCR4*, *FOXA2*, *GATA6*, *SOX17* and pluripotency genes (*OCT3/4*, *SOX2*) in the parental cell lines (GCT27, NCCIT, and NT2/D1), YST‐like cells in vitro at d8 of differentiation, and respective tumor tissues isolated from YST‐like xenografts were analyzed. Three biological replicates were analyzed in technical triplicates each. *GAPDH* and *Beta‐Actin* served as housekeepers and for data normalization. Standard deviations are given above each bar.

We xenografted YST‐like cells (bulk populations of GCT27, NCCIT, NT2/D1; *n* = 3 each) into the flank of nude mice to demonstrate that the in vitro differentiated YST‐like cells also resemble a YST in vivo (Figure 2A). The cell line 1411H, which resembles YST in vivo, was included as a control.^40^ After reaching a size of 1.5 cm^3^, tumors were isolated (Figure 2A,B). In immunohistochemical staining, all YST‐like tumor tissues were focally positive for the YST markers SOX17 and FOXA2 and showed a YST‐like morphology in hematoxylin and eosin staining (Figure 2C). Additionally, populations positive for EC / pluripotency markers (OCT3/4^+^, SOX2^+^) were found, while other populations were SOX17^−^ and FOXA2^−^ (Figure 2C). All tumor cells were SALL4^+^, a general GCT marker (Figure 2C, SOX2 inlay). Typically for YST, the 1411H cells presented as SOX17^+^, FOXA2^+^, SALL4^+^, OCT3/4^−^, and SOX2^−^ (Figure 2C). By qRT‐PCR analysis, upregulation of *APOA1*, *CXCR4*, *SOX17*, *FOXA2*, and *GATA6* expression has been validated in YST‐like cells in vitro and in vivo, while *OCT3/4* and *SOX2* were downregulated (Figure 2D). Similar deregulations in gene expression were found in xenografted 2102EP cells (*n* = 1) (Figure S2A). These analyses demonstrated that the in vitro differentiated YST‐like cells form a mixed GCT including EC and YST‐like populations in vivo.

### 3.5 Molecular analysis of YST formation at the single cell level

**FIGURE 3:**
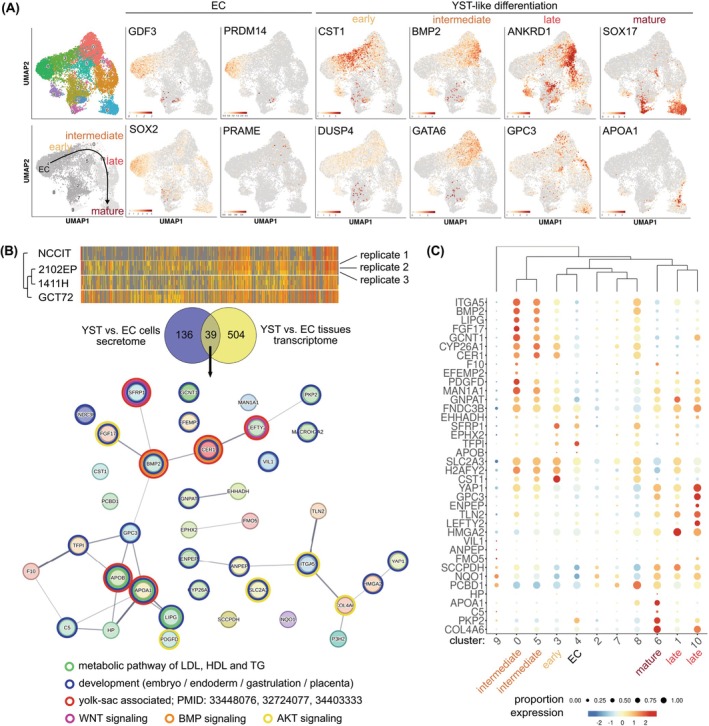
(A) UMAP clustering of YST‐like GCT27 and NCCIT cells based on 11 different unique transcriptional signatures (clusters 0 – 10) (left side) including an illustration of the dynamics of YST formation (black arrow) and expression analysis of marker genes indicative for EC and YST cells, identifying early, intermediate, late and mature YST‐like cells. scRNAseq has been performed in biological duplicates. (B) A heatmap including hierarchical clustering compares the secretomes of the EC cell lines 2102EP and NCCIT to the YST cell lines 1411H and GCT72. Each analysis was performed in technical triplicates. A Venn diagram summarizes factors highly expressed in YST tissues versus EC tissues and secreted by YST cells versus EC cells. The interaction of these factors and linked molecular functions was predicted by the STRING algorithm. (C) Bubble plot illustrating the expression of factors expressed and secreted by YST compared to EC cells/tissues in each transcriptional cluster identified by scRNAseq.

To further decipher the molecular mechanisms of YST formation from EC, we performed scRNAseq of the pluripotent EC cell lines NCCIT and GCT27 after treatment with SACF (at d8) and identified 11 different transcriptional signatures by UMAP analysis (Figure 3A). We found expression of typical EC markers, like *GDF3, PRDM14*, and *SOX2*, mainly in cluster (c) 4, which we postulate to represent a population of (so far) undifferentiated EC cells (Figure 3A). In line, these cells did not express the SEM marker *PRAME* (Figure 3A). By screening for the expression of typical YST markers, we identified several clusters representing YST‐like cells in different, partly overlapping, stages of the differentiation process (early [c3; c5, c0; e.g., *CST1* ^+^, *DUSP4* ^+^], intermediate [c0, c5, c10; e.g. *BMP2* ^+^, *GATA6* ^+^], late [c1; e.g., *ANKRD1* ^+^, *GPC3* ^+^], and mature YST cells [c6, c9, c7, c2; e.g., *SOX17* ^+^]) (Figure 3A). At early stages, both EC / pluripotency and YST markers could be found (e.g., partly overlapping *GDF3 / SOX2* and *CST1 / DUSP4* expression), while at later stages until mature YST‐like cells, the expression of EC / pluripotency factors diminished and mainly YST factors were found (Figure 3A). Next, we used scRNAseq to analyze the EC cell line 2102EP, which has a limited potential to differentiate compared to the other EC cell lines. Similar to the pluripotent NCCIT and GCT27 cell lines, we found the expression of early (*CST1*, *DUSP4*), intermediate (*BMP2*, *GATA6*), late (*ANKRD1*, *GPC3*), and mature (*SOX17*, *APOA1*) YST markers after SACF application (Figure S2B). Thus, both nulli‐ and pluripotent EC cells are able to transit into a YST‐like cell fate. All genes exclusively found in each cluster for all analyzed cell lines are given in Data S1B,C.

To further confirm the YST‐like nature of the differentiated NCCIT and GCT27 cells, we re‐evaluated and correlated gene expression to secretome data of YST and EC tissues and cell lines (Figure 3B; Data S1D).^8^, ^41^ First, we identified genes upregulated in expression in YST tissues compared to EC tissues (FC > 4).^8^ Next, we correlated these transcripts to the secretome level, i.e., proteins secreted the YST cell line GCT72 compared to the EC cell lines 2102EP and NCCIT to identify 39 factors increased in expression in and secreted by YST cells compared to EC cells (Figure 3B; Data S1D).^41^ Among these 39 factors were typical YST factors described in previous studies, like APOA1, CST1, and GPC3 as well as WNT (SFRP1), BMP (BMP2, CER), NODAL (LEFTY1/2), and AKT (PDGFD, SLC2A3, ITGA5, COL4A6, and FGF17) signaling molecules as well as lipoprotein metabolism (APOA1, APOB, and LIPG) associated factors (Figure 3B).^8^, ^42^, ^43^ Many of these factors (24 of 39) could be linked to developmental processes typical for yolk‐sac (YS) formation, like embryonal / endodermal / placenta development and gastrulation (Figure 3B). Now, we screened for expression of these factors in our scRNAseq data of NT2/D1 and GCT27. A bubble blot including hierarchical clustering demonstrated that regarding expression of these factors, c3 and c4 as well as c0 and c5 grouped together, reflecting undifferentiated (c4) and early (c3), and intermediate YST‐like cells (c0, c5), respectively (Figure 3C). Additionally, c6, c1, and c10 grouped together, reflecting late (c1, c10) and mature (c6) YST‐like cells (Figure 3C). Thus, these factors expressed in and secreted by YST tissues and cells are also present in the various stages of differentiation of the YST‐like cells.

### 3.6 Identification of SOX17 and FOXA2‐binding sites

**FIGURE 4:**
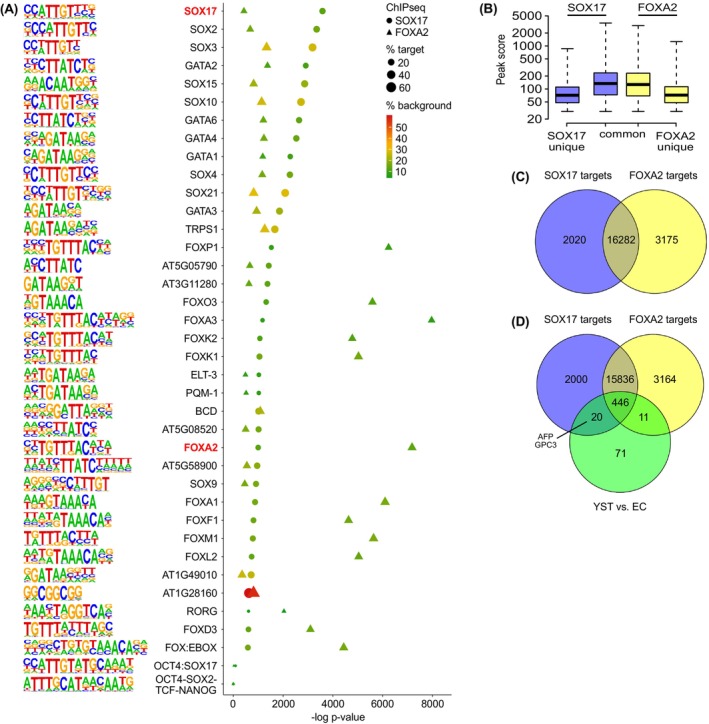
(A) Binding motifs and enrichment plot of sequences bound by SOX17 and FOXA2 as detected by ChIPseq analysis performed in technical triplicates in the EC cell lines NT2/D1 and GCT27. (B) A boxplot illustrates the average peak score of SOX17 and FOXA2 ChIPseq data for peaks unique to or common to SOX17 and / or FOXA2. (C) A Venn diagram illustrates the number of target sequences bound by SOX17 or FOXA2 or both in GCT72 and 1411H cells as found by ChIPseq analysis. (D) A Venn diagram illustrates the number of SOX17 and FOXA2 target sequences in genes overexpressed in YST compared to EC tissues (YST vs. EC).

We demonstrated that SOX17 and FOXA2 are key players of the YST fate determination.^8^ To identify binding sites of SOX17 and FOXA2, we performed a SOX17 / FOXA2‐ChIPseq analysis in 1411H and GCT72 YST cells. We first validated suitability of used antibodies to enrich SOX17 / FOXA2 from cell lysates of GCT72 and 1411H cells by western blotting after incubation with magnetic beads coupled to related antibodies (Figure S3A). Next, we performed the ChIPseq analysis (Data S1E,F). Both transcription factors showed a similar genome‐wide occupancy mainly around transcription start sites and promotors (FOXA2‐ChIP 53.09 %; SOX17‐ChIP 57.06 %) (Figure S3B,C). A HOMER‐based known motif enrichment plot demonstrated that with very high significance the SOX17 motif was enriched in the SOX17‐ChIP samples and the FOXA2 motif in the FOXA2‐ChIP samples, demonstrating that the ChIP analysis technically performed well (Figure 4A). Furthermore, among the most enriched motifs in the SOX17‐ and FOXA2‐ChIP were further SOX (SOX2/3/4/9/10/15/21), GATA (GATA1/2/3/4/6), and FOX (FOXA1/A3/D3/F1/K2/K1/L2/M1/O3/P1) motifs (Figure 4A). In the SOX17‐ChIP, enrichment of the OCT4 / SOX17 (compressed motif) or the OCT4 / SOX2 / TCF / NANOG (canonical) motif could not be observed (Figure 4A).^44^, ^45^ Next, we identified all binding sites within annotated genes solely bound by SOX17 (*n* = 2020) or FOXA2 (*n* = 3175) or both (*n* = 16,282) (FDR corrected *q*‐value < .001), demonstrating that the majority of genes bound by SOX17 was also bound by FOXA2 (Figure 4B,C; Figure S3D; Data S1G). Examples of peak histograms of SOX17 / FOXA2 binding to targets identified by the ChIPseq are given in Data S1H. We compared the set of SOX17 and FOXA2 targets to the genes shown to be differentially expressed between YST and EC (*n* = 548; FC > 2) (Figure 4D).^8^ Of the 548 differentially expressed genes, 456 (83.2 %) were bound by both, SOX17 and FOXA2, while 20 were bound exclusively by SOX17 (including *AFP* and *GPC3*) and 11 by FOXA2 (Figure 4D; Data S1G). The STRING algorithm predicted interaction between the commonly bound factors (including SOX17 and FOXA2) that could be linked to molecular features like “cell differentiation,” “endoderm formation,” “FOXA2 pathway,” and “WNT / TGF‐beta / MAPK signaling,” so basically YST‐associated processes (Figure S4A).

### 3.7 Acquisition of cisplatin resistance accompanies YST formation

**FIGURE 5:**
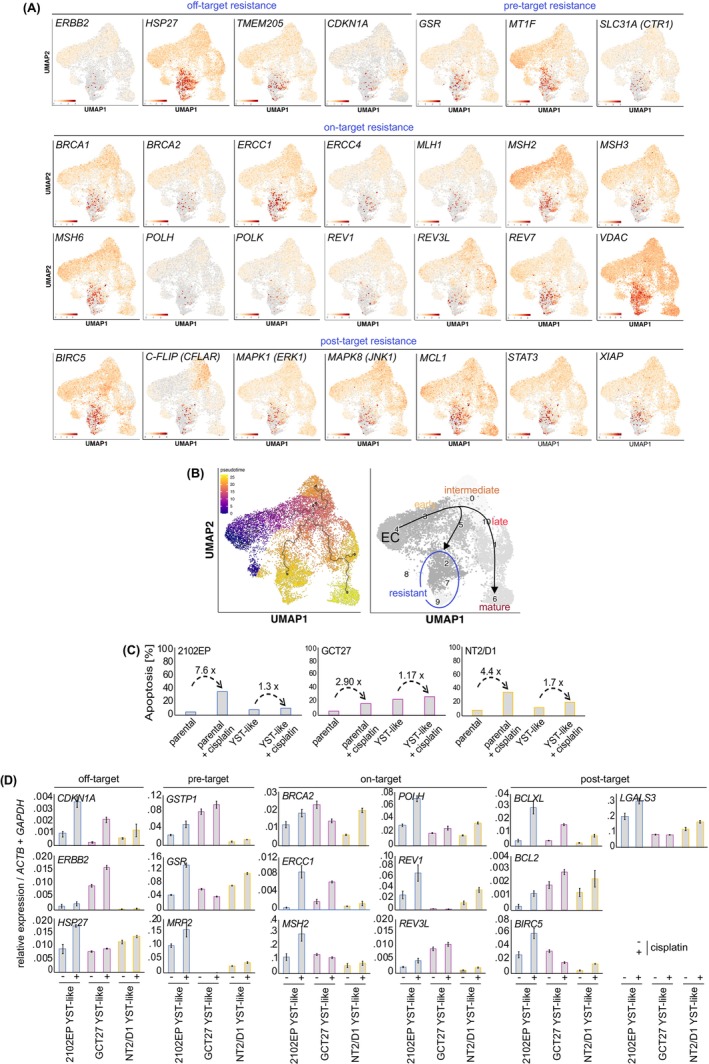
(A) Identification of cisplatin resistance signatures in YST‐like GCT27 and NCCIT cells at d8 based on expression of known resistance factors stratified by Galluzzi et al. into off‐, pre‐, on‐, and post‐target effectors. (B) UMAP clustering of YST‐like GCT27 and NCCIT cells based on 11 different unique transcriptional signatures and illustration of the dynamics of YST formation and of a cisplatin‐resistant subpopulation based on a pseudotime analysis with color‐coded temporal kinetics from purple to yellow. (C) Annexin V / PI‐based flow cytometry measurement of apoptosis induction in YST‐like and parental 2102EP, NT2/D1, and GCT27 cells 48 h after treatment with the LD_50_ values of cisplatin. (D) Relative gene expression of factors involved in cisplatin resistance in cisplatin‐treated (LD_50_, 48 h) YST‐like and parental EC cells. All samples were normalized to the respective solvent control. Three biological replicates (2102EP, GCT27, NT2/D1) were analyzed in technical triplicates each. *GAPDH* and *Beta‐Actin* served as housekeepers and for data normalization. Standard deviations are given above each bar.

We hypothesized that the formation of a YST is an escape mechanism for GCT under therapy, thus we asked if the differentiation of EC cells into the YST lineage is accompanied by an increased cisplatin resistance. Therefore, we questioned the expression of known cisplatin resistance factors (Galluzzi factors) in our scRNAseq data (Figure 5A).^46^ We found considerably high expression of various factors associated with the different Galluzzi groups (i.e., off‐target: *ERBB2*, *HSP27*, *TMEM205*; pre‐target: *GSR*, *MT1F*, *SLC31A* (CTR1); on‐target: *ERCC1*, *MLH1*, *MSH2*, *MSH3*, *MSH6*, *POLH*, *POLK*, *REV1*, *REV3L, REV7, VDAC*; post‐target: *BIRC5*, *C‐FLIP*, *MAPK1* (ERK1), *MAPK8* (JNK1), *MCL1*, *STAT3*, and *XIAP*) mainly in c9, c2, and c7 (but not in c6) overlapping with *SOX17* expression (Figure 5A,B, right side). Thus, we assume that a subset of YST‐like cells acquired cisplatin resistance (Figure 5B, right side). In nullipotent 2102EP, we also found expression of the analyzed Galluzzi factors, either in the late and / or mature YST‐like clusters or throughout all detected cells (Figure S2C). To narrow down the developmental origin of the resistant cell population, we performed pseudotime analysis of the scRNAseq data, demonstrating that the resistant and non‐resistant mature YST‐like cells putatively developed during differentiation and in parallel from a group of cells resembling intermediate YST‐like cells (c0, c5) (Figure 5B). Thus, cisplatin resistance was acquired in cells already resembling YST and not in undifferentiated EC cells. In line, treatment of EC cells with CHIRON, ActivinA, or FGF2 alone had rather negligible effects on the expression of Galluzzi factors as shown by qRT‐PCR analysis (Figure S4B). We identified all genes differentially (or exclusively) expressed during the formation of the mature resistant and non‐resistant YST‐like cells (DataS1I,J). Among the genes exclusively expressed in the resistant subpopulation were factors associated with ABC transporters, DNA repair, and methyltransferase activity (DNA and histones) (based on the DAVID algorithm), representing further genes putatively involved in mediating cisplatin resistance and modifying the epigenetic landscape of the resistant YST‐like cells (Data S1J).

To confirm that the upregulation of Galluzzi factors affects the response to cisplatin, we differentiated 2102EP, NT2/D1, and GCT27 cells into the YST lineage and treated the cells from d7 on with the LD_50_ of cisplatin (2102EP: 9.06 μM; GCT27: 8.21 μM; NT2/D1: 4.90 μM), as determined by XTT viability assays (Figure S2D). As controls, undifferentiated and cisplatin‐treated EC cells were included. Subsequently, we measured induction of apoptosis by Annexin V / PI‐based flow cytometry. Cisplatin caused a 7.6‐ / 2.9‐ / 4.4‐fold increase in the percentage of apoptotic cells in undifferentiated 2102EP / NT2/D1 / GCT27 cells, while only a 1.3‐ / 1.2‐ / 1.7‐fold increase has been measured in YST‐like cells (Figure 5C). A qRT‐PCR analysis validated that cisplatin treatment of YST‐like cells led to a further increase in the expression of selected Galluzzi factors in a cell line‐dependent manner (Figure 5D).

## 4 DISCUSSION

**FIGURE 6:**
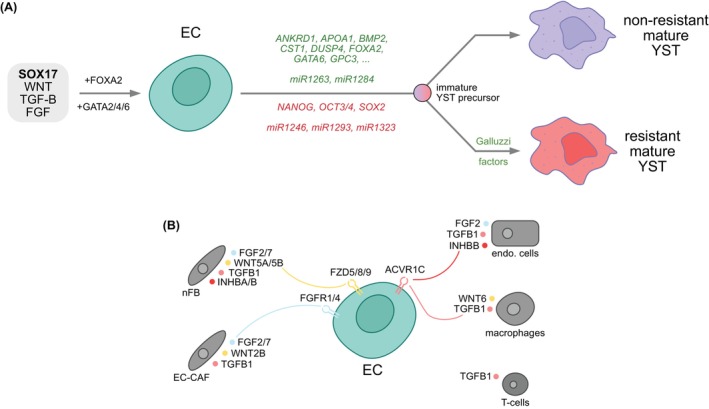
Graphical models summarize the most important findings of this study. (A) YST formation from EC is initiated by combined activity of SOX17 and the WNT, TGF‐beta / Activin, and FGF pathway, leading to upregulation of YST‐associated marker genes and microRNAs (labelled in green) as well as downregulation (of pluripotency factors (labelled in red). Formation of a YST is an escape mechanism of EC under therapy, presumably resulting in formation of a mature cisplatin sensitive and resistant subpopulation from a common developmental immature YST‐like precursor. (B) Overview of chemokines secreted by various cells of the tumor microenvironment and stimulating corresponding receptors on EC cells, subsequently supporting differentiation into the YST lineage. CAF = cancer associated fibroblast; endo. cells = endothelial cells; nFB = normal fibroblast.

In this study, we demonstrated that the orchestrated induction of SOX17 and activation of the WNT, TGF‐beta / Activin, and FGF signaling pathways drive EC into the YST lineage in vitro (Figure 6). Additionally, microRNAs seem to influence the formation of a YST cell fate (Figure 6). This experimentally validates that pluripotent EC are able to differentiate into the extra‐embryonic lineage, reflecting their developmental origin of a totipotent (primordial) germ cell. Within the eight days of differentiation, a mixed GCT population arises, consisting of an undifferentiated EC population as well as early, intermediate, and mature YST cell populations, including stable *SOX17* expression (Figure 6). These populations are also detectable after xenotransplantation of the YST‐like cells into nude mice.

As shown previously, the transcription factors SOX2 and SOX17 play a bivalent role in GCT, i. e., *SOX2* is expressed in EC, while *SOX17* is expressed in SEM and extra‐embryonic YST.^1^ While SOX2 and SOX17 can maintain an undifferentiated cell fate in EC (pluripotent) and SEM (latent pluripotent), SOX17 is also able to switch to a differentiation‐inducing function, which seems to occur in EC after forced induction of *SOX17* expression, which stabilizes over time in the YST‐like cells, while *SOX2* expression is considerably reduced (this study).^44^, ^45^, ^47^, ^48^ Nevertheless, our study shows that for differentiation of EC cells into the YST lineage, SOX17 stimulation alone seems not to be sufficient, but it rather depends on the co‐stimulation of the WNT and TGF‐beta / Activin pathway, while stimulation of FGF signaling is not necessary, but positively influences cellular viability during differentiation. In line, Xu et al.^49^ found high activity of WNT signaling in pediatric and adult GCT, especially in YST. Thus, YST are the only GCT where high WNT activity and *SOX17* expression can be detected in parallel (in SOX17^+^ SEM no WNT activity was found), putatively allowing for the establishment and maintenance of a YST cell fate.^50^, ^51^

We screened The Cancer Genome Atlas GCT cohort for the expression of WNT, FGF, and TGF‐beta / Activin signaling‐associated receptors; compared to SEM, we found high expression of related receptors in non‐SEM, demonstrating that especially EC are responsive to related ligands (via *FGFR1*/*4*, *FZD5*/*8*/*9*, *GPC1*/*3*/*4*/*5*, *ACVR1C*) (Figure S5A; Figure 6). We propose that the ligands are secreted by cells of the surrounding microenvironment. Indeed, by re‐evaluating RNAseq data from previous studies of our laboratory, we found expression of WNT, TGF‐beta / Activin, and FGF effector molecules in fibroblasts (mainly FGF2/7, WNT5A/5B, TGFB1, INHBA/B), EC‐associated cancer‐activated fibroblasts (EC‐CAF; *FGF2*/*7*, *WNT2B*, *TGFB1*), endothelial cells (HUVEC; *FGF2*, *TGFB1, INHB*), T‐cells (JURKAT; *TGFB1*) and M2‐macrophages (THP‐1‐M2; *WNT6*, *TGFB1*) (Figure S5B; Figure 6).^41^, ^52^ Taken together, EC cells are responsive to WNT, TGF‐beta / Activin, and FGF effector molecules provided by various cell types of the microenvironment, suggesting that the cross‐talk of EC with the microenvironment is an important step in YST formation (Figure 6). Additionally, WNT signaling has been shown to regulate the *SOX17* expression during extra‐embryonic endoderm formation (i.e. the YS). Thus, activation of WNT signaling by the microenvironment (or in vitro by CHIRON) might be responsible for the stabilization of the *SOX17* expression, which is still highly detectable eight days after infection in vitro, or after weeks in vivo.

Furthermore, we postulated that FOXA2 is a key driver of the YST fate.^7^, ^53^ Our ChIPseq suggested that many SOX17 targets and YST‐associated factors are also bound by FOXA2, confirming that both factors are involved in establishing and maintaining a YST cell fate. The regulatory hierarchy of SOX17 and FOXA2 could not be determined from ChIPseq data, but after SACF application and xenotransplantation of YST‐like cells, upregulation of FOXA2 could be observed, suggesting that FOXA2 rather acts in concert or even downstream of SOX17. Based on our ChIPseq data, the GATA factors, especially GATA4 and GATA6, may interact with SOX17 and FOXA2, which is in line with our previous findings showing that *GATA4* and *GATA6* were highly upregulated in YST tissues compared to EC and were predicted to interact with SOX17 and FOXA2.^8^ Thus, the molecular functions of SOX17 and FOXA2 might be unfolded with the help of GATA factors during YST formation, which nevertheless needs to be proven functionally (Figure 6). Further, our ChIPseq data suggest that the established YST marker genes *AFP* and *GPC3* are directly regulated by SOX17, but not FOXA2.

Mackinlay et al.^43^ and Markouli et al.^54^ described the in vitro differentiation of human (h) pluripotent stem cells / embryonic stem cells (hESC, hPSC) into the extra‐embryonic endoderm / YS lineage. Since EC cells malignantly resemble hESC, we hypothesize that the molecular mechanisms driving YS or YST formation from hESC or EC, respectively, should be quite similar. Indeed, in CHIRON and ActivinA stimulated YS from hESC / hPSC, YST tissues, and YST‐like cells, upregulation of endodermal factors, like *AFP*, *APOA1*, *ANKRD1*, *CST1*, *FOXA2*, *GATA6*, and *SOX17*, and downregulation of the pluripotency factors *NANOG*, *OCT3/4*, and *SOX2* could be measured (Figure 6).^43^, ^54^ Markouli et al.^54^ also demonstrated that sustained activation of WNT (CHIRON) and BMP (BMP4) signaling impairs differentiation of hESC to the definitive endoderm and eventually drives the cells towards the extra‐embryonic mesoderm, which could be rescued by inhibition of WNT (XAV‐939) and BMP (NOGGIN) signaling. In our hands, treating NCCIT EC cells with XAV‐939 or NOGGIN rather reduced the expression of YST‐associated factors or had only minor effects. Thus, during the formation and maintenance of a YST‐like fate, a sustained activity of WNT seems not to be disadvantageous. In line, Mukherjee et al.^55^ found that SOX17 together with active WNT signaling drives hPSC into the endoderm lineage. Additionally, Mukherjee et al.^56^ showed that SOX17 recruits beta‐CATENIN to enhancer regions of genes regulating endoderm formation during Xenopus gatrulation. These data further support the importance of the interaction of SOX17 and WNT signaling in the YST cell fate.

Besides these molecular changes, the transition of EC cells into the YST lineage was accompanied by changes in microRNA expression. Of the six upregulated and 14 downregulated microRNAs highlighted by Wruck et al.,^8^ five were also found upregulated and nine downregulated in YST versus EC tissues / cells by microRNAseq and were deregulated in YST‐like cells. Thus, these microRNAs are putatively involved in the formation and maintenance of a YST cell fate. Among them, microRNAs upregulated in YST targeting (as identified by Wruck et al.^8^ using TargetScan 7.2) the germ cell and pluripotency network (*TFAP2C* [*miR1263*], *DND1* [*miR1284*], *BCAT1* [*miR1263*], *KLF4* [*miR1263*]) and microRNAs downregulated in YST targeting YST‐associated factors also found deregulated in this study, like *AFP* (*miR1323*), *ANKRD6* (*miR1246*), *BMP2* (*miR1246*), *CST1* (*miR1293*), *DUSP4* (*miR1293*) *FOXA2* (*miR1246*), GATA6 (*miR1246*), and SOX17 (*miR1246*).

We also detected a cluster of cells showing transcriptional features of cisplatin‐resistant GCT, i.e., increased expression of the Galluzzi factors, mainly pre‐, on‐, and off‐target factors, suggesting that the formation of a YST is accompanied by the acquisition of therapy resistance (Figure 6). This is confirmed by our pseudotime analysis of scRNAseq data, where the resistant and non‐resistant mature YST populations develop from cells already in the process of a cell fate switch from EC to YST but not from undifferentiated EC cells (Figure 6). Thus, YST development is indeed an escape mechanism for GCT under therapy. Deducing from our results, targeting SOX17 or the WNT, TGF‐beta / Activin, or FGF signaling pathways in combination with cisplatin might be a promising way to block the escape route of GCT under therapy, i.e., formation of a YST.^53^

In summary, our study deciphered the molecular mechanisms forcing EC to differentiate into the YST lineage in detail, i. e., by activation of SOX17 and the WNT, TGF‐beta / Activin, and FGF signaling pathways. Additionally, SOX17 and FOXA2 may act in concert with GATA factors to induce the cell fate switch, which is accompanied by the acquisition of cisplatin resistance. Our findings confirm that the formation of a YST is an escape mechanism for GCT under therapy. Thus, GCT patients should be screened for YST elements under therapy to identify patients at risk of developing therapy resistance. In the future, our results should be used to deduce novel therapeutic concepts that block the formation of a YST under therapy in combination with standard therapeutics, like cisplatin.

## AUTHOR CONTRIBUTIONS

**Mara Kotthoff:** Methodology; validation; formal analysis; investigation; writing – original draft; visualization; writing – review and editing. **Margaretha A. Skowron:** Methodology; validation; formal analysis; investigation; visualization. **Felix Bremmer:** Investigation; resources; funding acquisition; writing – review and editing. **Fatma Parmaksiz:** Investigation. **Pia Kretschmer:** Investigation. **Alexa Stephan:** Validation; investigation. **Alexander Fichtner:** Investigation. **Tobias Lautwein:** Software; methodology; formal analysis; data curation; visualization. **Katharina Raba:** Investigation. **Janina Fuß:** Investigation. **Karl Köhrer:** Software; formal analysis; resources; data curation. **Daniel Nettersheim:** Conceptualization; methodology; formal analysis; resources; data curation; writing – original draft; visualization; writing – review and editing; project administration; supervision; funding acquisition.

## FUNDING INFORMATION

D. Nettersheim, Margaretha A. Skowron and F. Bremmer were supported by funding of the “German Cancer Aid” (Translational Oncology Collaborative Scientific Project; 70115997). Additionally, D. Nettersheim was financially supported by the “Center for Integrated Oncology Aachen Bonn Cologne Düsseldorf” (CIO ABCD). F. Bremmer was supported by the “Wilhelm Sander‐Stiftung” (2016.041.1/.2/.3). This work was supported by the DFG “Research Infrastructure NGS_CC” (project 407495230) as part of the “Next Generation Sequencing Competence Network” (project 423957469).

## CONFLICT OF INTEREST STATEMENT

No conflict of interest has been declared.

## ETHICS STATEMENT

The ethics committees of the Medical Faculty of Heinrich Heine University Düsseldorf (EC‐HHU‐D) the University Medicine Göttingen (EC‐UMG) raised no concerns on performing experiments using GCT tissues (vote 2024‐2879 to D.N.; vote 20/9/20 to F.B.). Additionally, no ethical concerns were raised by the EC‐HHU‐D regarding the use of GCT cell lines for in vitro experiments (2019‐783 to D.N.).

## Supporting information


Data S1.

Table S1.

Table S2.

Data S2.

## Data Availability

All data generated or analyzed during this study are included in the published article, and its supplementary data or can be requested from the corresponding author. scRNAseq, microRNAseq, and ChIPseq data have been uploaded to “Gene Expression Omnibus” (GEO; https://www.ncbi.nlm.nih.gov/geo/) (GSE241060, GSE254140, GSE276475). RNAseq data of fibroblasts, endothelial cells, T‐cells, macrophages, as well as cancer‐associated fibroblasts are also available via GEO (GSE195794, GSE229047). Previously published secretome data have been re‐analyzed in context of this study and is available via ProteomeXchange (https://www.proteomexchange.org) (PXD031329).^41^ Further information is available from the corresponding author upon request.
