# Supplementary material for: Induction of SOX17 with stimulation of WNT, TGF‐beta, and FGF signaling drives embryonal carcinomas into the yolk‐sac tumor lineage resulting in increased cisplatin resistance
Source: Int J Cancer. 2025 Mar 2;156(11):2210–24. doi: 10.1002/ijc.35385 (PMC11970549; doi:10.1002/ijc.35385)
Supplement: Supplementary file 4 — Data S2. [file IJC-156-2210-s004.pdf]

Induction of SOX17 with stimulation of WNT, TGF-beta and FGF signaling drives embryonal carcinomas into the yolk-sac tumor lineage resulting in increased cisplatin resistance

Mara Kotthoff, Margaretha A. Skowron, Felix Bremmer, Fatma Parmaksiz, Pia Kretschmer, Alexa Stephan, Alexander Fichtner, Tobias Lautwein, Katharina Raba, Janina Fuß, Karl Köhrer, Daniel Nettersheim

Table of Content

1. **Supplemental Material and Methods**

2. **Supplemental Figures**

3. **Data S1 (uploaded / provided as separate file)**
A) microRNA sequencing data of YST versus EC tissues/cell lines. B, C) scRNAseq data of B) GCT27 and NCCIT and C) 2102EP cells. D) Factors expressed in YST versus EC tissues and secreted by YST versus EC cell lines. E, F) SOX17- and FOXA2-ChIPseq data (averaged values of GCT72 (n=3) and 1411H cells (n=3); normalized to input controls (n=2). G) Evaluation of ChIPseq data with regard to individually and commonly bound target sequences by SOX17 and FOXA2. H) Examples of SOX17 and FOXA2 binding to YST-associated genes. I, J) Genes differentially expressed during formation of mature non-resistant (I) and resistant (J) YST-like cells. In (J), also genes exclusively expressed during formation of resistant YST-like cells are given, including a STRING interaction prediction and DAVID molecular function annotation.

4. **Table S1 (uploaded / provided as separate file)**
A) Cell lines, B) drugs, C) oligonucleotides and D) antibodies used in this study.

5. **Table S2 (uploaded / provided as separate file)**
Sequencing coverage and quality statistics for each microRNAseq (A), scRNAseq (B) and ChIPseq (C) sample.

Induction of SOX17 with stimulation of WNT, TGF-beta and FGF signaling drives embryonal carcinomas into the yolk-sac tumor lineage resulting in increased cisplatin resistance

Mara Kotthoff, Margaretha A. Skowron, Felix Bremmer, Fatma Parmaksiz, Pia Kretschmer, Alexa Stephan, Alexander Fichtner, Tobias Lautwein, Katharina Raba, Janina Fuß, Karl Köhrer, Daniel Nettersheim

Supplemental Material and Methods

Treatment of EC cells *in vitro*

For the treatment of EC cells with recombinant proteins or inhibitors, 4 *10^3 cells were seeded out into 6-well plates. The recombinant proteins or inhibitors were applied as given in Table S1 B (Table S1 B). The medium was refreshed every second a day for eight days in total. All analysed were performed in technical triplicates.

Production of lentiviral particles and SOX17 expression induction

To produce lentiviral particles (LVP) in eukaryotic HEK293-T cells, calcium phosphate precipitation was performed by using the 'Profection - Mammalian Transfection System' kit (Promega GmbH, Walldorf, Germany). Further, lentiviral plasmid DNA was used to enable packaging (psPAX2 #12260, Addgene, LGC Standards, Teddington, UK) and formation of the viral envelope (pMDG.2 #12259, Addgene). For induction of endogenous *SOX17* expression, the lentiSAMv2 vector (#75112, Addgene) containing a guideRNA (5'-GTGGGGTTGGACTGGGACGT-3') targeting *SOX17* (lentiSAMv2-*SOX17*) was used. For production of LVP allowing for the induction of SOX17 via the CRISPR/dCas9-SAM system (SOX17-LVP), 7x10^6 HEK293-T cells were plated into 10 ml standard cell culture medium on 10 cm poly-L-lysine coated cell culture dishes and incubated at 37 °C overnight. On day one, the DNA to be transfected (psPAX2, pMD2.G, lentiSAMv2-*SOX17* or lentiMPHv2) was mixed with the 2 M calcium chloride solution provided in the kit, then transferred drop by drop into the reaction tube containing HEPES buffer with continuous vortexing and incubated at room temperature (RT) for 15 minutes (min). Afterwards, the medium of the HEK293-T cells was replaced by 5 ml of the pre-warmed 'Advanced Medium 2 %' (DMEM Advanced Medium, 2 % FBS, 1 % P/S, 1 % L-glutamine). To increase transfection efficiency, 25 mM chloroquine was added (1:1000) to the medium. The calcium phosphate-DNA transfection complex was added dropwise to the target cells and then incubated for 5 hours (h) at 37 °C. Afterwards, the medium was changed to 10 ml 'Advanced Medium 5 %' (DMEM Advanced Medium, 5 % FBS, 1 % P/S, 1 % L-glutamine) and incubated overnight at 37 °C. The next day, the medium was changed again to 7 ml 'Advanced Medium 5 %'. On day three, virus-containing cell culture supernatant was collected and stored at 4 °C. On day four, cell culture supernatant was collected according to the same procedure and added to the aliquot of the previous day. The virus suspension was filtered (0.45 µM) to remove cell debris and then frozen in 500 µl aliquots at -80 °C until usage.

Generation of stable lentiMPHv2$^+$ EC cell lines

To increase efficacy of endogenous *SOX17* induction by the CRISPR/dCas9-SAM system, EC cell lines stably expressing the MS2-P65-HSF1 activator helper complex (EF1a-MS2-p65-HSF1-2A-Hygro-WPRE; lentiMPHv2 plasmid, #89308, Addgene) were generated via transduction with MPHv2 LVP (2102EP-MPHv2$^+$, 833KE-MPHv2$^+$, GCT27-MPHv2$^+$, NCCIT-MPHv2$^+$, NT2/D1-MPHv2$^+$). For selection of MPHv2$^+$ cells, 250 µg/ml Hygromycin B (Carl Roth, Karlsruhe, Germany) was added to standard culture medium for two weeks and refreshed after each passaging. As controls, parental cell lines were included.

RNA and protein isolation

mRNA was isolated using the ‚RNeasy mini kit‘ (Qiagen, Hilden, Germany) according to the manufacturer's instructions. RNA was eluted in 35 µl H_2O. Total RNA from cells or tumor tissues was isolated by the ‘Phenol/guanidine-based QIAzol Lysis Reagent’ according to the manual (Qiagen). The tumour tissues were minced and lysed in 1 ml of ‘QIAzol Lysis Reagent’ (Qiagen) in a homogeniser. Total RNA was eluted in 35 (cells) or 50 - 100 µl (tissues) of H_2O. Nucleic acid concentrations and purities were measured by the Nanodrop 2000 photo-spectrometer (Thermo Fisher Scientific, Schwerte, Germany) (260/280 nm; 260/230 nm). For protein isolation, cells were lysed for 30 min on ice in 50 - 100 ul x RIPA buffer (‚Stock solution 10 x RIPA‘; Cell Signaling Technology, Frankfurt am Main, Germany) containing ‘cOmplete Protease Inhibitor Cocktail’ (Merck). The lysates were centrifuged at 12,000x g at 4 °C for 15 min and the protein containing supernatants were collected. Protein concentrations was determined by the ‘Pierce BCA Protein Assay Reagent Kit’ (Thermo Fisher Scientific) and measured on ‘iMark Microplate Reader’ (Bio-Rad, Feldkirchen, Germany).

Quantitative RT-PCR

The protocol for complementary DNA (cDNA) synthesis and quantitative reverse transcriptase polymerase chain reaction (qRT-PCR) was performed as published previously[1,2]. For each cDNA synthesis, 1000 ng of RNA were used and reverse transcribed using ‘Maxima H Minus Reverse Transcriptase (200 U/µl)‘, ‚Oligo(dT)18 Primer‘ (0.5 µg/µl), RT buffer (5 x), dNTP mix (10 mM) and ‚RiboLock RNase Inhibitor‘ (40 U/µl) (all Thermo Fisher Scientifics). cDNA was diluted 1:17 with RNAse-free water (Sigma-Aldrich, Steinheim, Germany) to a final concentration of 50 ng/µl [2]. Each gene

expression analysis was performed in technical triplicates on 384-well plates using 7.34 ng of diluted cDNA and the SYBR-green based 'Luna Universal qPCR Master Mix' (New England Biolabs, Frankfurt am Main, Germany) on a 'CFX384 Touch Thermal Cycler' (Bio-Rad). For quality control, melt curve analyses were performed for each run. *ACTB* and *GAPDH* were used as housekeeping genes and for normalization. See Table S1 C for oligonucleotide sequences (Table S1 C).

qPCR analysis of microRNA expression

For cDNA synthesis, the instructions of the 'miRCURY LNA Starter Kit' (Qiagen) were followed using 100 ng of total RNA or 5 µl of purified microRNA. 0.5 µl 'UniSp6 RNA spike-in' were added to each sample. microRNA expression was analyzed using the 'miRCURY LNA miRNA SYBR Green PCR kit' (Qiagen) according to the instructions. 2 µl of cDNA was diluted in a 1:60 ratio and 3 µl of the diluted sample was added to 5 µl '2x miRCURY SYBR Green Master Mix', 1 µl 'Resuspended PCR primer mix' and 1 µl RNase-free H_2O (10 µl total volume). Each sample was analyzed in technical triplicates on a 'CFX384 Touch Thermal Cycler' (Bio-Rad). Primers for each microRNA were ordered as 'ready-to-use' solutions from Qiagen.

Immunohistochemistry

Immunohistochemical staining (IHC) was performed on 4 µm formalin-fixed paraffin-embedded (FFPE) tissue sections with a Dako Autostainer or the Dako Omnis system with the Dako EnVision FLEX+ detection system (Dako, Agilent Technologies, Waldbronn, Germany), as described previously [3,4]. Utilized antibodies are given in Table S1 D. Deparaffinisation, rehydration and heat-induced epitope-retrieval were carried out in one step with the 3-in-1 procedure buffer (Dako Target Retrieval Solution) at 97 °C with a PT Link Pre-Treatment Module (Dako, Agilent Technologies). Tissue samples were analysed with light microscopy after counterstaining with Meyer's haematoxylin for 8 min (Dako, Agilent Technologies).

Western Blot

For each performed immunoblot, 20 µg of each protein lysate were used. Sample preparation and western blotting was carried out as described previously [1,2]. Briefly, membranes were blocked in PBS + 1 % Tween-20 (PBS-T) + 5 % skimmed milk powder (Sigma-Aldrich) for 1 h at RT and then incubated with primary antibodies

overnight at 4 °C. Vinculin was used as loading control. Species-specific HRP-conjugated secondary antibodies were incubated for 2 h at RT. For chemilumincent-based protein visualization, the ‘Clarity™ Western ECL-Substrate’ (Bio-Rad) and the ‘Chemidoc Imaging System’ (Bio-Rad) were used. For immunoprecipitation (IP) prior to western blotting, 10 µg of primary antibody/IP were coupled to 1.5 mg Dynabeads M-270 Epoxy beads (Thermo Fisher Scientific) at 37 °C for 24 h according to the manufacturer's instructions. Afterwards, antibody-coupled beads were incubated with 1 mg of protein lysate at 4 °C for 1 h. Elution was performed in 22 µl 1 x SDS buffer (diluted 4 x SDS buffer containing 30 % glycerol, 12 % SDS, 150 mM Tris base in H_2O, pH 7.0) at 95 °C for 5 min on a rocking platform at 300 rpm. VINCULIN was used as housekeeper and loading control. See Table S1 D for antibody details (Table S1 D).

Apoptosis assay via flow cytometry

Cells were collected after trypsinization at 37 °C for up to 5 min, washed once with ‘1 x Annexin V binding buffer‘ (Miltenyi Biotec, Bergisch Gladbach, Germany) and then stained with Annexin V-FITC and propidium iodide (PI) for following flow cytometric based analysis as described previously [5–7]. All apoptosis assays were performed on ‘MACS Quant Analyzer 10’ (Miltenyi Biotec) and analysed by the ‘MACSQuantify V 2.11’ software (Miltenyi Biotec). See Table S1 D for antibody details (Table S1 D).

Flow cytometry-based cell sorting

For fluorescence activated cell sorting (FACS) of $CXCR4^{-/+}$ cells, EC cells were differentiated into the YST lineage following our established protocol. Then, cells were trypsinized and collected. To stratify cells into $CXCR4^{-/+}$ populations, YST-like cells were stained by a PE-conjugated CXCR4 antibody (1:100) diluted in 40 µl FACS buffer (phosphate-buffered saline (PBS), pH 7.2, 0.5 % bovine serum albumin (BSA) and 2mM EDTA) for 15 min with shaking in the dark at RT. Cells were then washed with FACS buffer and re-suspended in 1 ml FACS buffer and kept on ice until FACS was performed. Cell sorting was conducted on the ‚MoFloTM XDP‘ cell sorter (Beckman Coulter, Krefeld, Germany) equipped with near UV (375 nm), blue (488 nm), red (640 nm) lasers, 13 detectors and ‘Summit‘ software (Beckman Coulter, v5.4.0.). The default configuration of the instrument was a 100 µm nozzle with 39 KHz sorting frequency, pressure 26 PSI and drop delay of 30. Cells were identified based on forward scatter (FSC-H) vs. side scatter (SSC-H), doublet exclusion (SSC-W vs. SSC-

H) and positivity and negativity of the CXCR4 PE signal. See Table S1 D for antibody details (Table S1 D).

XTT cell viability measurements

XTT cell viability assays have been performed as described previously [6]. $3x10^3$ cells were seeded out in technical quadruplicates in a volume of 50 µl into 96-well plates. For normalization, solvent controls were included. 24 - 96 h post application of drugs, XTT salt (494.93 µmol/L) and phenazine methosulfate (4.2 µmol/L) was applied to the cells. After 4 h of incubation at 37 °C, the absorbance was measured by the ‚iMark Absorbance Reader' (Bio-Rad; absorbance: 450 nm; reference: 655 nm). Half-lethal doses (LD_{50}) values were calculated by 'GraphPad Prism v8' (GraphPad Software, Inc, San Diego, CA, USA).

Xenotransplantation

Xenotransplantation was performed as described earlier [8,9]. Briefly, EC cell lines (GCT27-MPHv2$^+$, NCCIT-MPHv2$^+$, NT2/D1-MPHv2$^+$) were differentiated into the YST lineage following our established protocol. 1411H cells served as controls. Cells were trypsinized, counted and $1x10^7$ cells were re-suspended in 500 ml of Matrigel (BD, Heidelberg, Germany). The cells were injected into the flank of six-weeks-old, male CD1 nude mice (Charles River, Erkrath, Germany) (n=3 for each cell line) using a cold syringe. Tumor growth was followed by measuring the three-dimensional tumor size using a caliper and tumors were isolated when tumor size was around 1 cm^3. Tumor samples were divided lengthways; one half was fixed in 4 % paraformaldehyde and further embedded in FFPE for IHC staining and the other half was used for RNA isolation. Animal experiments were performed under license of the 'Landesamt für Natur und Umwelt - Nordrhein-Westfalen' (LANUV-NRW; AZ-84-02.04.2013-A430 to D. N.).

Corresponding literature

1. Nettersheim, D. *et al.* BMP Inhibition in Seminomas Initiates Acquisition of Pluripotency via NODAL Signaling Resulting in Reprogramming to an Embryonal Carcinoma. *PLoS Genet* **11**, (2015).
2. Skowron, M. A. *et al.* CDK4/6 inhibition presents as a therapeutic option for paediatric and adult germ cell tumours and induces cell cycle arrest and apoptosis via canonical and non-canonical mechanisms. *Br J Cancer* (2020) doi:10.1038/s41416-020-0891-x.
3. Fichtner, A. *et al.* Primary mediastinal germ cell tumours: an immunohistochemical and molecular diagnostic approach. *Histopathology* **80**, 381–396 (2022).
4. Fichtner, A. *et al.* The detection of isochromosome i(12p) in malignant germ cell tumours and tumours with somatic malignant transformation by the use of quantitative real-time polymerase chain reaction. (2020) doi:10.1111/his.14258.
5. Nettersheim, D. *et al.* A signaling cascade including ARID1A, GADD45B and DUSP1 induces apoptosis and affects the cell cycle of germ cell cancers after romidepsin treatment. *Oncotarget* **7**, 74931–74946 (2016).
6. Skowron, M. A. *et al.* The signal transducer CD24 suppresses the germ cell program and promotes an ectodermal rather than mesodermal cell fate in embryonal carcinomas. *Mol Oncol* **16**, 982–1008 (2022).
7. Söhngen, C. *et al.* CD24 targeting with NK-CAR immunotherapy in testis, prostate, renal and (luminal-type) bladder cancer and identification of direct CD24 interaction partners. *FEBS J* **290**, 4864–4876 (2023).
8. Burmeister, A. *et al.* Establishment and Evaluation of Dual HDAC/BET Inhibitors as Therapeutic Options for Germ Cell Tumors and Other Urological Malignancies. *Mol Cancer Ther* **21**, 1674–1688 (2022).
9. Müller, M. R. *et al.* Therapeutical interference with the epigenetic landscape of germ cell tumors: a comparative drug study and new mechanistical insights. *Clin Epigenetics* **14**, (2022).

Induction of SOX17 with stimulation of WNT, TGF-beta and FGF signaling drives embryonal carcinomas into the yolk-sac tumor lineage resulting in increased cisplatin resistance

Mara Kotthoff, Margaretha A. Skowron, Felix Bremmer, Fatma Parmaksiz, Pia Kretschmer, Alexa Stephan, Alexander Fichtner, Tobias Lautwein, Katharina Raba, Janina Fuß, Karl Köhrer, Daniel Nettersheim

Supplemental Figures

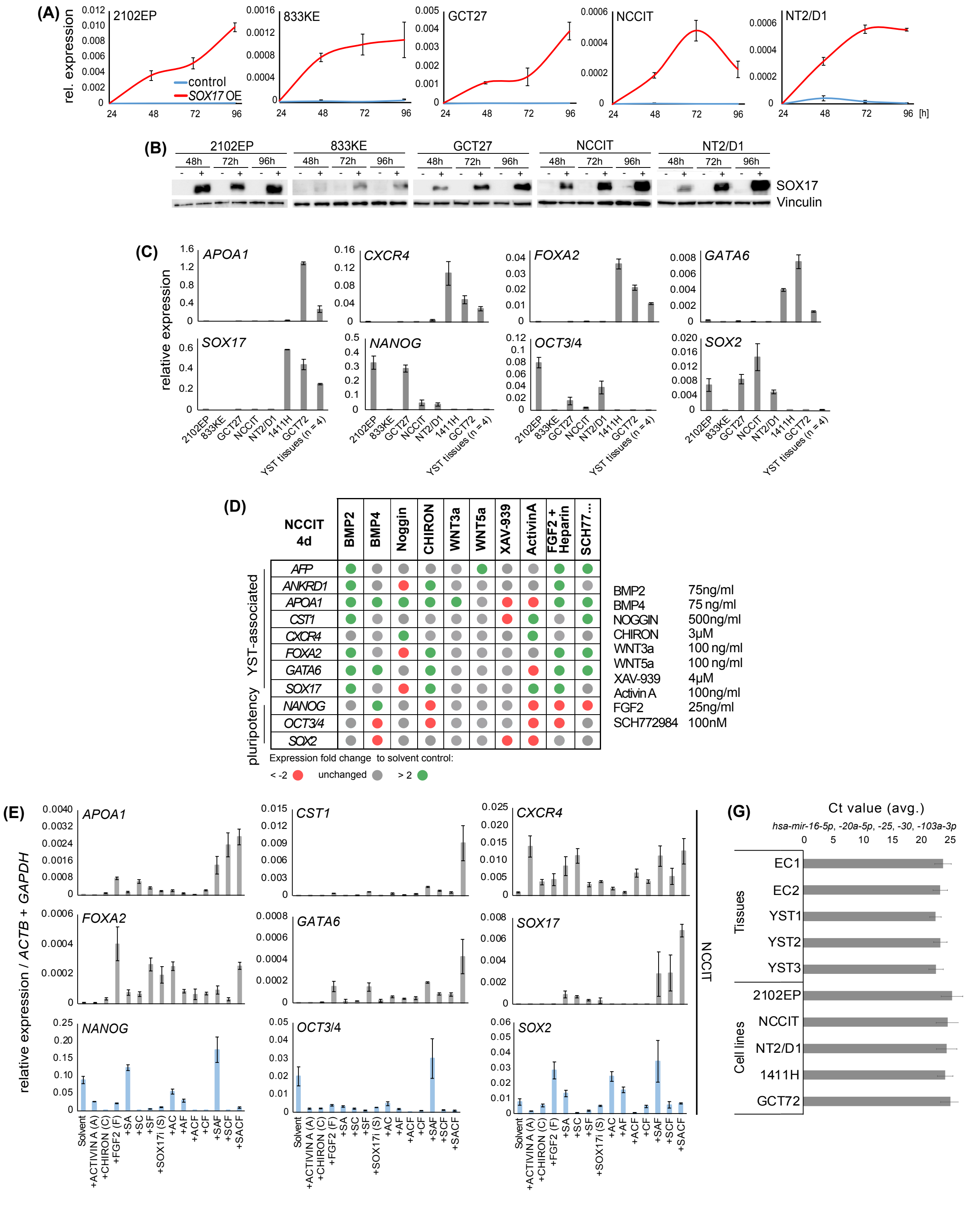

(F)

cell line	sample	cell count (8d) [cells/ml]	cell viability [%]	sorted YSTL cells [%]
2102EP	solvent control	$4.25x10^6$	98	CXCR4 (-) 7.21
	YSTL differentiated	$1.95x10^6$	96	CXCR4 (+) 6.86
NT2/D1	solvent control	$9.29x10^5$	98	CXCR4 (-) 9.01
	YSTL differentiated	$6.41x10^5$	96	CXCR4 (+) 6.49

Figure S1:

A) Relative gene expression of *SOX17* in EC cells (2102EP, 833KE, GCT27, NCCIT and NT2/D1) treated with SOX17-inducing LVPs (red line) and their solvent control treated parental cells (blue line) measured over 96 h. Each

analysis was performed in technical triplicates each. *GAPDH* and *Beta-Actin* served as house-keepers and for data normalization. Standard deviations are given above each bar.

B) Western blot analysis of SOX17 in EC cells (2102EP, 833KE, GCT27, NCCIT and NT2/D1) treated with SOX17-inducing LVPs (+) and their solvent control treated parental cells (-) over 96 h.

C) Basal gene expression intensities of YST-associated genes (*APOA1*, *CXCR4*, *FOXA2*, *GATA6*, *SOX17*) and pluripotency genes (*NANOG*, *OCT3/4*, *SOX2*) in EC cell lines (2102EP, 833KE, GCT27, NCCIT, NT2/D1), EC-YST-intermediate cells (1411H), YST cells (GCT72) and YST tissues (n=4). Each analysis was performed in technical triplicates each. *GAPDH* and *Beta-Actin* served as house-keepers and for data normalization. Standard deviations are given above each bar.

D) Graphical illustration of qRT-PCR analysis of changes in expression of YST- and pluripotency-associated factors in NCCIT cells at d4 after stimulation with indicated recombinant proteins or inhibitors. Each analysis was performed in technical triplicates each. *GAPDH* and *Beta-Actin* served as house-keepers and for data normalization.

E) Relative gene expression of YST-associated genes (*APOA1*, *CST1*, *CXCR4*, *FOXA2*, *GATA6*, *SOX17*) and pluripotency genes (*NANOG*, *OCT3/4*, *SOX2*) in NCCIT after treatment with the single, double, triple and quadruple combinations to optimize the conditions for establishing the YST-like differentiation protocol *in vitro*. Each analysis was performed in technical triplicates each. *GAPDH* and *Beta-Actin* served as house-keepers and for data normalization. Standard deviations are given above each bar.

F) Cell counts and viability of YST-like 2102EP and NT2/D1 cells at d8 of differentiation used for CXCR4$^{+/-}$ FACS.

G) Expression analysis of microRNAs *hsa-mir-16-5p*, *-20a-5p*, *-25*, *-30*, *-103a-3p* demonstrated a highly homogeneous expression in YST and EC tissues/cell lines.

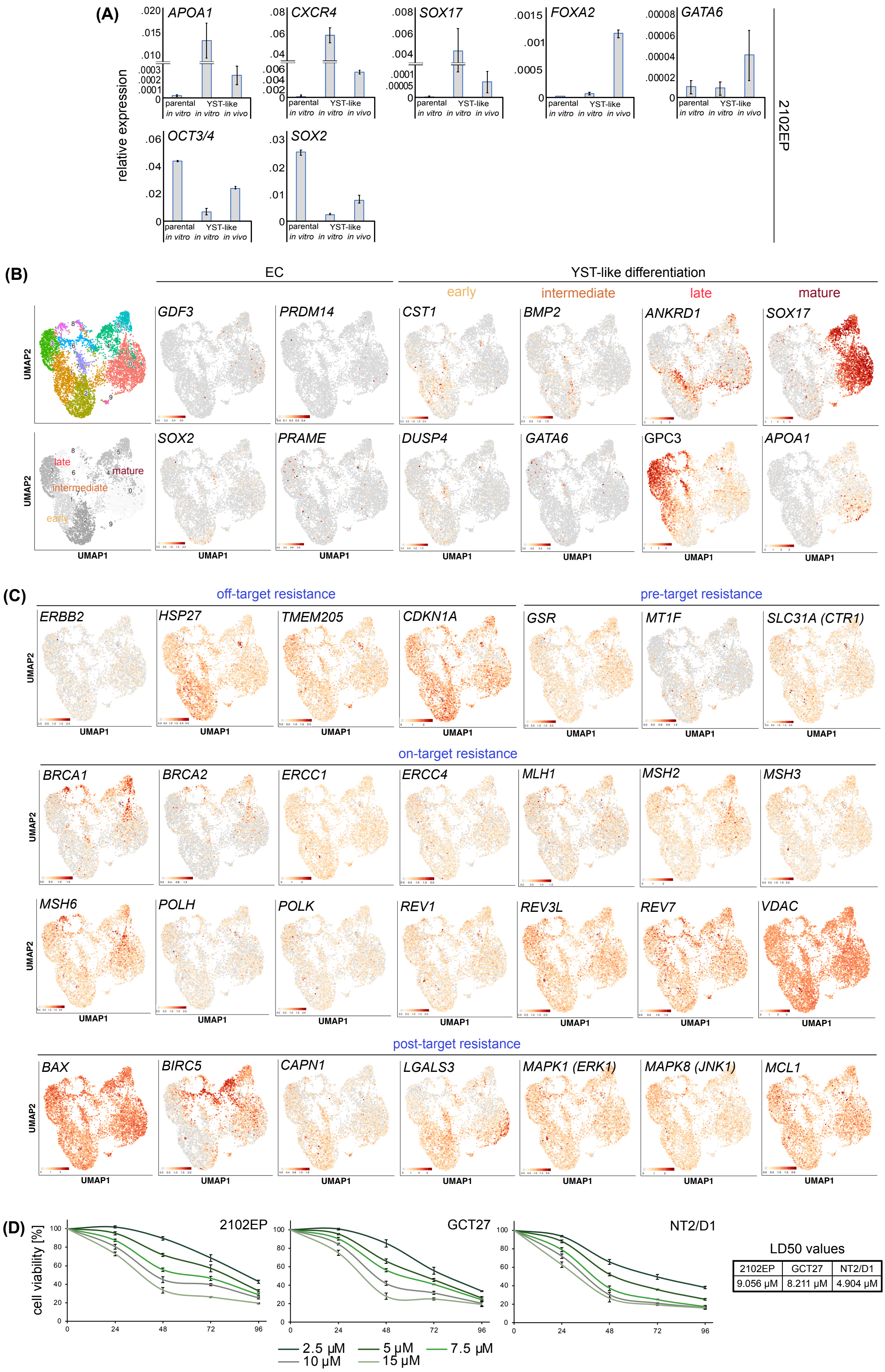
(A)
relative expression
APOA1
CXCR4
SOX17
FOXA2
GATA6
OCT3/4
SOX2
parental
YST-like
in vitro
in vivo
2102EP
(B)
EC
YST-like differentiation
early
intermediate
late
mature
GDF3
PRDM14
CST1
BMP2
ANKRD1
SOX17
SOX2
PRAME
DUSP4
GATA6
GPC3
APOA1
UMAP1
UMAP2
(C)
off-target resistance
pre-target resistance
ERBB2
HSP27
TMEM205
CDKN1A
GSR
MT1F
SLC31A (CTR1)
on-target resistance
BRCA1
BRCA2
ERCC1
ERCC4
MLH1
MSH2
MSH3
MSH6
POLH
POLK
REV1
REV3L
REV7
VDAC
post-target resistance
BAX
BIRC5
CAPN1
LGALS3
MAPK1 (ERK1)
MAPK8 (JNK1)
MCL1
(D)
cell viability [%]
2102EP
GCT27
NT2/D1
2.5 µM
5 µM
7.5 µM
10 µM
15 µM
LD50 values
2102EP | GCT27 | NT2/D1
9.056 µM | 8.211 µM | 4.904 µM

Figure S2:

A) Relative gene expression of YST-associated genes (*APOA1*, *CXCR4*, *FOXA2*, *GATA6*, *SOX17)* and pluripotency genes (*OCT3/4*, *SOX2*) in the parental cell line 2102EP, YST-like cells *in vitro* at d8 of differentiation and respective tumor tissues isolated from YST-like xenografts (n=1). * = samples related to 2nd y-axis. Each analysis was performed in technical triplicates each. *GAPDH* and *Beta-Actin* served as house-keepers and for data normalization. Standard deviations are given above each bar.

B) UMAP clustering of YST-like 2102EP cells based on 10 different, unique transcriptional signatures (clusters 0-9) (left side) and expression analysis of marker genes indicative for EC and YST cells, identifying early, intermediate, late and mature YST-like cells.

C) Identification of cisplatin resistance signatures in YST-like 2102EP cells at d8 based on expression of known resistance factors stratified by Galluzzi et al. into off-, pre-, on-, and post-target effectors.

D) XTT cell viability assays in 2102EP, GCT27 and NT2/D1 cells after treatment with various cisplatin concentrations from 2.5-15 µM. Data were used to calculate the half lethal (LD_{50}) doses. Each assay was performed in technical quadruplicates each. Standard deviations are given at each time point (24-96 h).

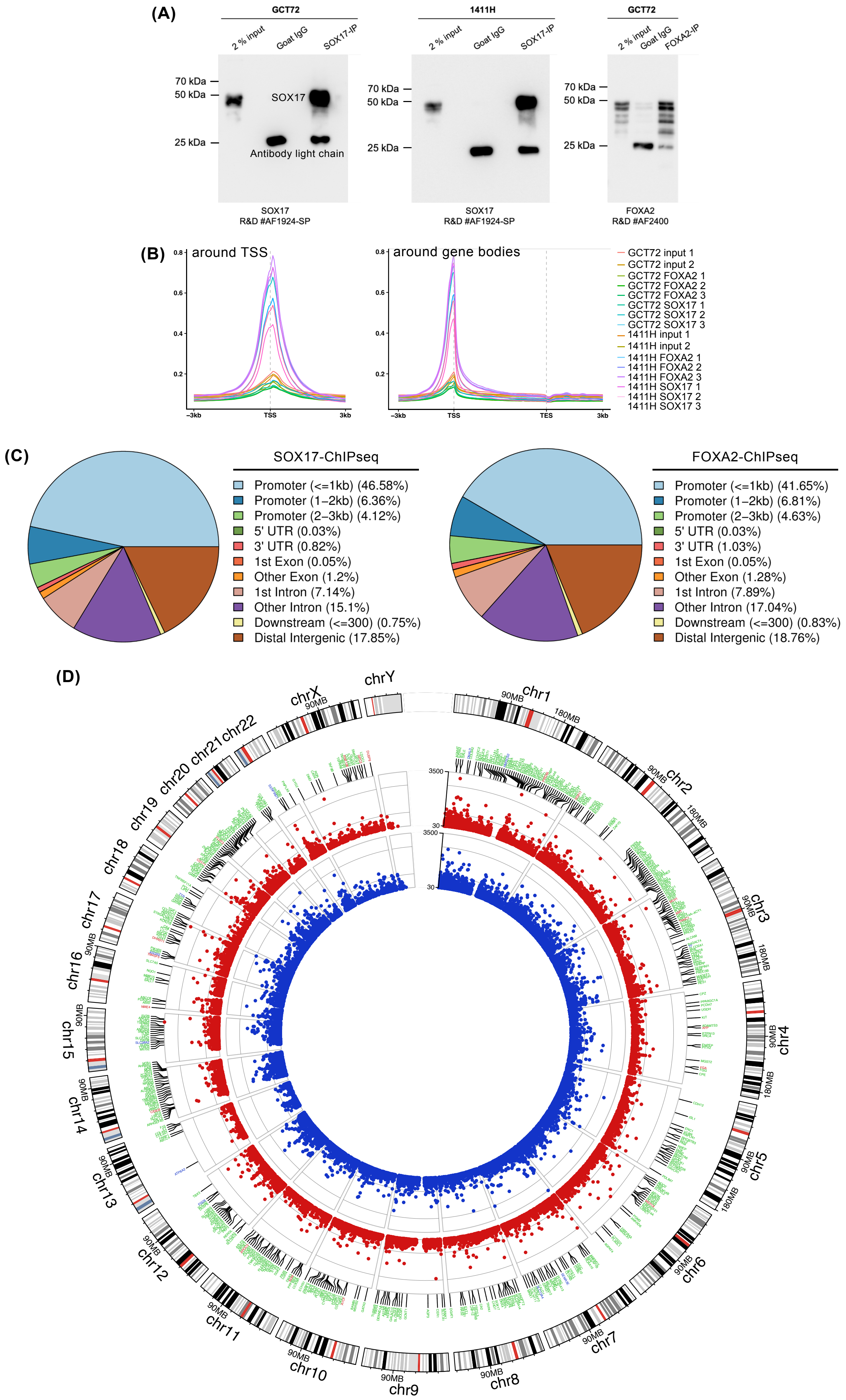

(A)
GCT72
2 % input
Goat IgG
SOX17-IP
70 kDa
50 kDa
25 kDa
SOX17
Antibody light chain
SOX17
R&D #AF1924-SP
1411H
2 % input
Goat IgG
SOX17-IP
70 kDa
50 kDa
25 kDa
SOX17
R&D #AF1924-SP
GCT72
2 % input
Goat IgG
FOXA2-IP
70 kDa
50 kDa
25 kDa
FOXA2
R&D #AF2400
(B)
around TSS
0.8
0.6
0.4
0.2
–3kb
TSS
3kb
around gene bodies
0.8
0.6
0.4
0.2
–3kb
TSS
TES
3kb
GCT72 input 1
GCT72 input 2
GCT72 FOXA2 1
GCT72 FOXA2 2
GCT72 FOXA2 3
GCT72 SOX17 1
GCT72 SOX17 2
GCT72 SOX17 3
1411H input 1
1411H input 2
1411H FOXA2 1
1411H FOXA2 2
1411H FOXA2 3
1411H SOX17 1
1411H SOX17 2
1411H SOX17 3
(C)
SOX17-ChIPseq
Promoter (<=1kb) (46.58%)
Promoter (1–2kb) (6.36%)
Promoter (2–3kb) (4.12%)
5' UTR (0.03%)
3' UTR (0.82%)
1st Exon (0.05%)
Other Exon (1.2%)
1st Intron (7.14%)
Other Intron (15.1%)
Downstream (<=300) (0.75%)
Distal Intergenic (17.85%)
FOXA2-ChIPseq
Promoter (<=1kb) (41.65%)
Promoter (1–2kb) (6.81%)
Promoter (2–3kb) (4.63%)
5' UTR (0.03%)
3' UTR (1.03%)
1st Exon (0.05%)
Other Exon (1.28%)
1st Intron (7.89%)
Other Intron (17.04%)
Downstream (<=300) (0.83%)
Distal Intergenic (18.76%)
(D)
chr1
chr2
chr3
chr4
chr5
chr6
chr7
chr8
chr9
chr10
chr11
chr12
chr13
chr14
chr15
chr16
chr17
chr18
chr19
chr20
chr21
chr22
chrX
chrY
90MB
180MB
3500
30

Figure S3:

A) Immunoprecipitation of SOX17 or FOXA2 followed by western blotting in GCT72 and 1411H cells to verify suitability of used antibodies to precipitate the proteins of interest for ChIPseq.

B) Histograms showing binding preferences of SOX17 and FOXA2 around the transcription start sites (TSS) and around gene bodies).

C) Pie charts summarizing binding preferences of SOX17 and FOXA2 across various genomic regions.

D) A Circos diagram illustrates distribution of common (green) and unique SOX17 (blue) / FOXA2 (red) binding sites in annotated genes across the genome.

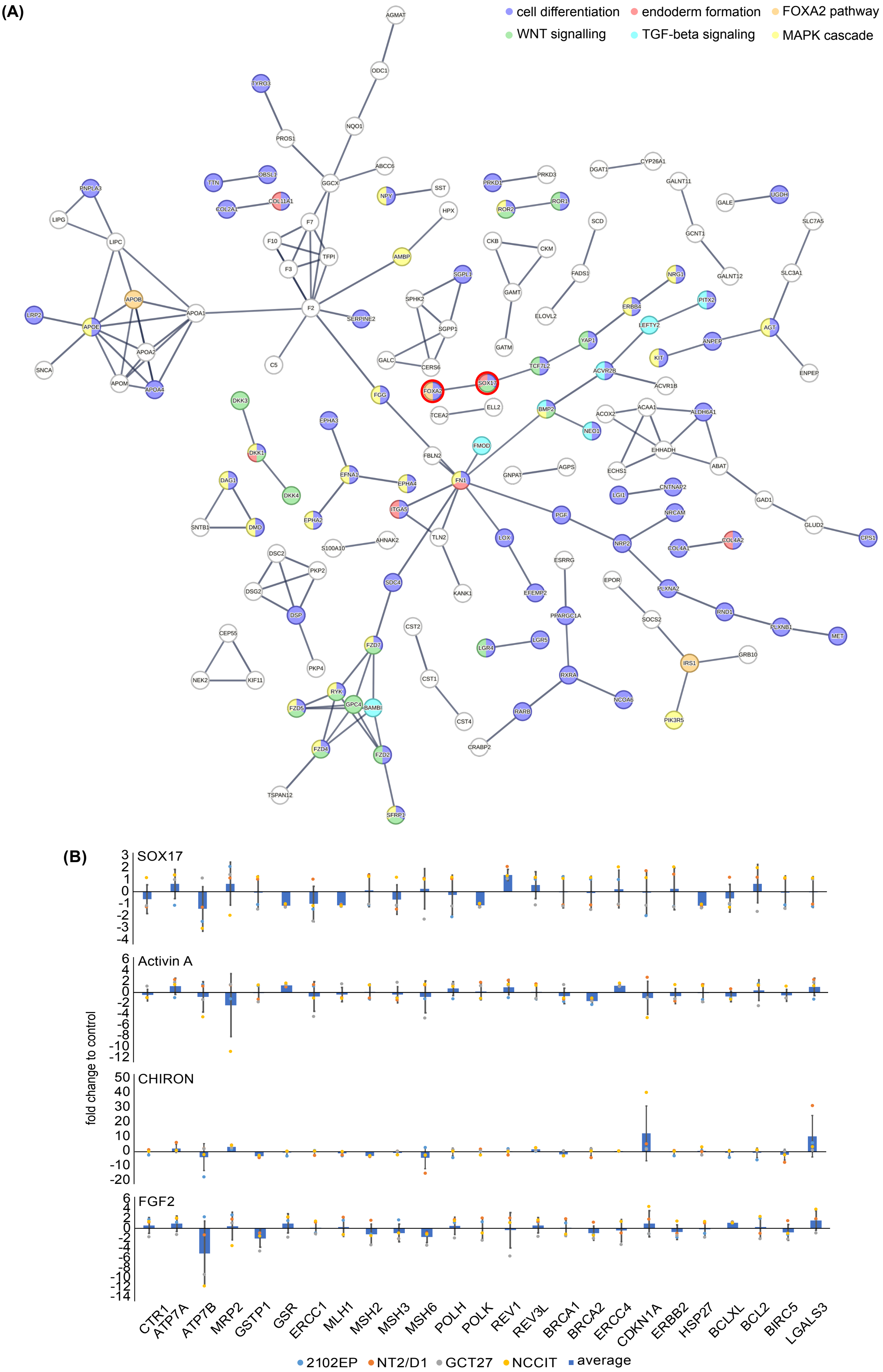
(A)
cell differentiation
endoderm formation
FOXA2 pathway
WNT signalling
TGF-beta signaling
MAPK cascade
(B)
SOX17
Activin A
CHIRON
FGF2
fold change to control
CTR1
ATP7A
ATP7B
MRP2
GSTP1
GSR
ERCC1
MLH1
MSH2
MSH3
MSH6
POLH
POLK
REV1
REV3L
BRCA1
BRCA2
ERCC4
CDKN1A
ERBB2
HSP27
BCLXL
BCL2
BIRC5
LGALS3
2102EP
NT2/D1
GCT27
NCCIT
average

Figure S4:

A) The STRING algorithm including GO analysis predicts protein-interactions and related molecular functions within the set of 446 genes bound by SOX17 and FOXA2 and overexpressed in YST versus EC tissues.

B) qRT-PCR analysis of expression of Galluzzi factors in EC cells after individual induction of SOX17 or treatment with ActivinA, CHIRON or FGF2 for 8d. Four biological replicates (2102EP, NT2/D1, GCT27, NCCIT) were analyzed in technical triplicates each. *GAPDH* and *Beta-Actin* served as house-keepers and for data normalization. Standard deviations are given above each bar.

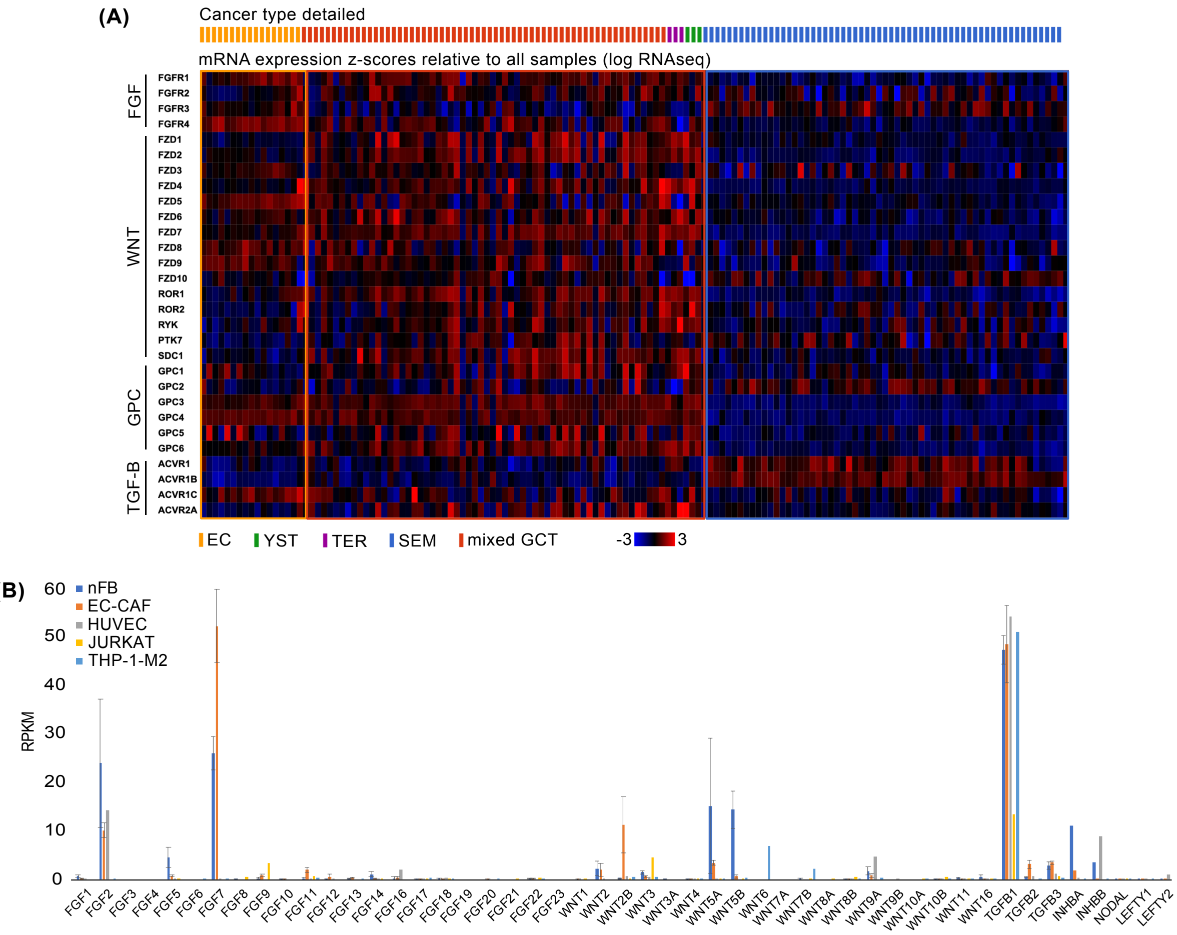

Figure S5:

A) cBioPortal-based analysis of expression of FGF-, WNT- and TGF-beta signaling-related receptors in GCT tissues (TCGA cohort).

B) RNA sequencing data (RPKM values) of expression of various signaling pathway effector molecules in normal fibroblasts (nFB; n=7 biological replicates), EC-associated cancer-activated fibroblasts (EC-CAF; n=3 biological replicates), endothelial cells (HUVEC; n=1), T-cells (JURKAT; n=1) and M2-macrophages (THP-1-M2; n=1).

Table S1:

A) Cell lines, B) drugs, C) oligonucleotides and D) antibodies used in this study.

Table S2:

Sequencing coverage and quality statistics for each microRNAseq (A), scRNAseq (B) and ChIPseq (C) sample.

Data S1:

A) microRNA sequencing data of YST versus EC tissues/cell lines. B, C) scRNAseq data of B) GCT27 and NCCIT and C) 2102EP cells. D) Factors expressed in YST versus EC tissues and secreted by YST versus EC cell lines. E, F) SOX17- and FOXA2-ChIPseq data (averaged values of GCT72 (n=3) and 1411H cells (n=3); normalized to input controls (n=2). G) TOP25 HOMER motif enrichment results of SOX17- and FOXA2-ChIPseq data. H) Evaluation of ChIPseq data with regard to individually and commonly bound target sequences by SOX17 and FOXA2. I, J) Genes differentially expressed during formation of mature non-resistant and resistant YST-like cells. In J), also genes exclusively expressed during formation of resistant YST-like cells are given, including a STRING interaction prediction and DAVID molecular function annotation.
